# TurboID Screening and Functional Analysis of the Interactome of the Broad-Spectrum Antiviral Protein Soybean Resistance Cluster 7 (SRC7)

**DOI:** 10.3390/biology15211949

**Published:** 2026-10-29

**Authors:** Zhuo Bao, Pengfei Diao, Wei Shao, Anar Bao, Aminna Bai, Hada Wuriyanghan, Lele Li

**Affiliations:** 1 School of Life Science, Inner Mongolia University, Hohhot 010030, China; nmbzhuo@163.com (Z.B.); shaowei1995812@163.com (W.S.); anarbao@163.com (A.B.); 15661061905@163.com (A.B.); 2 Key Laboratory of Herbage & Endemic Crop Biology, Ministry of Education (IMU), Hohhot 010070, China; 3 North Campus, Yantai Engineering & Technology College, Yantai 264006, China; lkzxdpf@163.com

**Keywords:** immune signaling, proximal candidate proteins, resistance protein, SRC7, TurboID screening

## Abstract

Plant viral diseases cause severe yield losses in global crop production. SRC7, a novel soybean TIR-NBS-BSP-type resistance (R) protein, confers broad-spectrum antiviral immunity against multiple viruses including soybean mosaic virus (SMV), tobacco mosaic virus (TMV), potato virus Y (PVY), and plum pox virus (PPV). Its TIR-NBS domain (SRC7^TN^) mediates full antiviral resistance, making it an ideal bait for screening immune-related proximal candidate proteins. In this study, TurboID-based proximity labeling (PL) was applied to profile the SRC7^TN^ proximal candidate proteins in *N. benthamiana*. We identified 262 high-confidence proximal candidate proteins, which were mainly enriched in plant immune signaling, redox regulation, protein modification and nuclear-related pathways. Among them, 21 candidates were functionally characterized, revealing multiple host factors contributing to SRC7-dependent antiviral immunity. Eleven interactions were confirmed via bimolecular fluorescence complementation (BiFC), and two nucleoside/nucleotide hydrolase-family proteins MZN14.5 and RIHA were subjected to further interaction-domain analysis. Collectively, this work constructs a high-confidence SRC7^TN^ proximal-interaction network and highlights critical host factors in SRC7-mediated broad-spectrum antiviral immunity. It also validates TurboID’s utility for screening for NLR-proximal candidate proteins in heterologous plant systems and provides insights into R-protein-governed plant antiviral defense.

## Simple Summary

Plant viral infections lead to large-scale crop yield losses worldwide. Certain plant resistance proteins can help crops fight against a wide range of viruses. One soybean protein named SRC7 provides strong protection against many plant viruses. A specific fragment of this protein is enough to activate antiviral defense and was used in this study to find other plant proteins that work together with it. We used a protein-labeling tool in experimental *Nicotiana benthamiana* plants to search for partner proteins. We found hundreds of candidate proteins and studied a subset of them. Several of these partners support plant antiviral defense. We further confirmed some protein-to-protein connections and analyzed two key proteins in greater detail. Our work builds a map of SRC7^TN^-proximal candidate proteins for this resistance fragment. It shows which host plant proteins take part in virus resistance. It also proves that this labeling tool works well for finding protein partners. These findings improve our understanding of how plants resist viruses and may support future crop-breeding efforts.

## 1. Introduction

Plant viruses cause significant yield losses in major crops. Plants evolve multilayered immune systems to counteract the pathogen attack. While RNA interference (RNAi) is regarded as a general antiviral mechanism, most plant viruses encode silencing suppressors which dampen RNAi by targeting the components of the RNAi pathway [1]. In parallel with RNAi, plants also possess pathogen-associated molecular pattern (PAMP)-triggered immunity (PTI) which is triggered by pattern recognition receptors localized on the plasma membrane. In addition, plants also develop a second layer of immunity called effector-triggered immunity (ETI), which is a specific response aiming to recognize the pathogen effector proteins that generally suppress host PTI [2]. ETI is often triggered by host-encoded resistance (R) proteins. Most of the R proteins belong to a large family of proteins harboring both a central nucleotide-binding site (NBS) domain and C-terminal leucine-rich repeat (LRR) domains and therefore are called NBS-LRR (NLR) proteins [3]. N-termini of plant NLR-type R proteins generally possess either TIR (toll/interleukin-1 receptor) or CC (coiled coil) domains, therefore they are termed as TNL (TIR-NBS-LRR) or CNL (CC-NBS-LRR) proteins respectively [4].

The establishment of plant R-protein-mediated signaling requires many other host partners. These partners directly or indirectly cooperate with R proteins to trigger immune responses. Traditional genetic analysis identified many host genes that are required for *R* gene signaling. In *Arabidopsis thaliana*, the TIR-NBS-class R protein RLM3 confers resistance to necrotrophic fungi like *Leptosphaeria maculans* depending on the upstream *R* gene *RLM1*. RLM1 acts as a key upstream regulator, supporting RLM3-mediated callose deposition, and genetic evidence confirms the two *R* genes play a role in the same immune pathway [5]. In *N. benthamiana*, TNL-type R proteins such as Roq1 (recognizing bacterial effectors XopQ/HopQ1) and RPP1 (recognizing oomycete effector ATR1) trigger immunity, which relies on the helper CNL-type R protein NRG1 and the lipase-like protein EDS1. Mutations in NRG1 completely eliminate the hypersensitive response (HR) and disrupt pathogen resistance, while EDS1 physically associates with NRG1 via its NBS and LRR domains and acts upstream of NRG1 to sustain TNL-mediated signaling [6]. The function of the *N* gene was impaired in transgenic *N. benthamiana* lines harboring the tobacco-class R gene *N* when virus-induced gene silencing (VIGS) targeted tobacco homologous genes *Rar1*, *EDS1* and *NPR1*/*NIM1*-like genes. This impairment was specifically characterized by the loss of HR, systemic spread of TMV (tobacco mosaic virus), and increased accumulation of viral RNA. The transcript levels of these genes were markedly reduced, thereby demonstrating that *Rar1*, *EDS1* and *NPR1*/*NIM1*-like genes are indispensable regulatory components in the *N* gene-mediated resistance pathway against TMV [7]. The CNL-type *R* gene *RPS2* confers resistance to *Pseudomonas syringae* pv. *tomato* DC3000 with the avirulence gene AvrRpt2, while a TRV (tobacco rattle virus)-based VIGS assay employed in transgenic *A. thaliana* lines harboring *RPS2* confirmed that *RIN4*, *RAR1*, *HSP90*, or *NDR1* play a crucial role in the *RPS2*-dependent resistance pathway. In contrast, VIGS-mediated silencing of *EDS1* and *SGT1b* participating in other *R* gene-mediated resistance pathways exerted no influence on *RPS2*-mediated resistance to *Pst.* DC3000 [8].

Genetic screening does not always identify direct interactors of R proteins, as the genes uncovered by such approaches often function in downstream signaling rather than engaging in direct physical interaction with the R protein itself. Therefore, the methods enabling large-scale identification of direct interactors are essential. In this regard, the yeast two-hybrid (Y2H) system has been widely employed. For example, Pto, which functions as an R protein by recognizing the pathogen effector AvrPto, was used as bait in Y2H screens. This led to the identification of three transcription factors, Pti4, Pti5, and Pti6, as well as the kinase Pti1, as interacting proteins. Among these, Pti4, Pti5, and Pti6 specifically bind to cis-elements present in the promoters of pathogenesis-related (*PR*) genes. Additionally, the tobacco ethylene-responsive element binding protein EREBP-2 was also confirmed to interact with Pto [9]. In another example, the tomato CNL-type R protein I-2, which confers resistance to the fungal pathogen *Fusarium oxysporum*, was used in Y2H screens with its LRR domain as bait. This led to the identification of protein phosphatase 5 (PP5) as an interacting partner. Subsequent screens revealed that two cytosolic heat shock protein 90 (HSP90) family members interact with PP5. HSP90 and PP5 bind to distinct regions of the I-2 LRR domain, and HSP90 is essential for I-2 function [10].

Despite its widespread application, the yeast two-hybrid (Y2H) system has intrinsic limitations. These include the inability to fully recapitulate endogenous protein interactions that occur in the plant cellular environment, overrepresentation of highly abundant housekeeping genes from cDNA libraries, a propensity for redundant results, and strong dependence on cDNA library quality. To address these constraints, alternative in planta screening methods have been developed. Affinity purification coupled with mass spectrometry (AP-MS) is one such approach [11,12]. In recent years, enzyme-catalyzed PL has also emerged as a powerful approach to overcome these limitations [13,14,15]. PL techniques were initially developed in animal systems and subsequently adapted for use in plants [16,17,18]. The core principle involves the fusion of a bait protein to an enzyme that catalyzes the covalent modification of neighboring proteins in vivo. These modified proteins can then be enriched by affinity capture and identified by mass spectrometry, enabling the mapping of proteins in close proximity to the bait during its functional engagement. Candidate interactors are inferred based on enrichment levels and statistical significance relative to controls [14,19,20]. A major advance in PL technology for plant research is TurboID, an engineered biotin ligase derived from directed evolution of *Escherichia coli* BirA. TurboID exhibits high enzymatic activity even at the relatively low temperatures optimal for plant growth, facilitating its rapid adoption in model plant systems [21,22].

For instance, the fusion of TurboID with the tobacco NLR protein N, combined with quantitative proteomics in *N. benthamiana*, identified multiple regulators of N-mediated immunity. Among these, the E3 ubiquitin ligase UBR7 was found to directly associate with the TIR domain of N and to negatively regulate N-mediated resistance to TMV [23]. In another study, TurboID fused to the guard-cell-specific transcription factor FAMA, along with an added nuclear localization signal, enabling the identification of numerous nuclear interactors, including known stomatal regulators and previously uncharacterized proteins [14]. TurboID has also been applied to investigate plasma-membrane-associated protein complexes in *N. benthamiana* and *A. thaliana*. This approach successfully identified both known and previously unknown interactors of the TPLATE complex, a membrane-associated protein assembly difficult to be characterized by AP-MS due to poor solubility of its components [19]. Collectively, TurboID-based PL offers several advantages for studying plant protein interactions: (1) it captures interactions in real time under physiologically relevant conditions; (2) it facilitates analysis of dynamic interaction networks during complex biological processes; (3) it overcomes technical barriers associated with studying membrane-associated proteins; and (4) it exhibits high sensitivity for detecting weak or transient interactions [24]. These attributes establish TurboID-based PL as a broadly applicable and powerful tool for elucidating protein interaction networks in plant biology.

Previously, we identified a novel soybean TIR-NBS-BSP-type R gene, *SRC7*, which exhibits several previously unreported features, including broad-spectrum viral resistance and a non-canonical BSP domain, and plays crucial roles in plant antiviral immunity. Specifically, SRC7 confers robust resistance to multiple viruses across different families, including soybean mosaic virus (SMV), potato virus Y (PVY), plum pox virus (PPV), and TMV. Its TIR domain is both necessary and sufficient for initiating immune signaling, while the NBS domain enhances this signaling activity in a cis-dependent manner and mediates essential nuclear oligomerization [25]. Notably, *N. benthamiana* possesses a complete immune pathway that supports SRC7-mediated immune cascades, including salicylic acid (SA) signaling, nuclear oligomerization, and downstream HR, and allows rapid assessment of antiviral activity through transient co-expression with viral infectious clones, making it an ideal experimental system for SRC7-related studies. Given that *Glycine max* is recalcitrant to efficient transient expression and high-throughput functional screening, while *N. benthamiana* has been firmly established as the universal heterologous model for studying NLR-mediated immunity, protein interactomes, and PL [14,21,23], we performed TurboID-based screening in this system. Notably, multiple landmark studies on plant NLR interactomes have successfully used *N. benthamiana* as the heterologous host for TurboID, including the well-known N protein-mediated antiviral immunity [14]. The conserved immune machinery including EDS1-NRG1 modules, redox regulation, and nuclear signaling is fully functional in *N. benthamiana* and supports SRC7-triggered immunity. Therefore, the heterologous system is genetically valid and methodologically well-established for dissecting the SRC7^TN^ proximal candidate proteins. In this work, we employed TurboID-based proximity labeling to identify host proteins proximal to SRC7^TN^, and functionally characterized several host factors contributing to SRC7-dependent antiviral immunity. This study provides new insights into the molecular basis of TIR-NBS-mediated plant antiviral immunity.

## 2. Materials and Methods

### 2.1. Plant Material and TurboID-Related Constructs

The *N. benthamiana* plants were grown in a greenhouse at 22–26 °C with a 16 h/8 h (light/dark) photoperiod. Plants were used for transient expression assays at 3–4 weeks of age.

For the construction of pCAMBIA1300-SRC7^TN^-TurboID-3×HA (TTH), a TurboID fragment was generated by PCR using the TurboID-His6_pET21a plasmid (Addgene, Watertown, MA, USA, #107177) as a template; the resulting fragments, SRC7^TN^ fragments and 3×HA fragments were ligated into *ScaI*/*KpnI* double-digested pCAMBIA1300 vector by homologous recombination (Vazyme, Nanjing, China, Cat#C115). pCAMBIA1300-NLS-eGFP-TurboID-3×HA (NGTH) was constructed using a similar strategy except that the eGFP fragment was cloned from pEarleyGate103 vector (Addgene #140007). SRC7^TN^ and NLS (PKKKRKV) have been described in previous studies [25].

For the cloning of several candidate genes, total RNA was isolated from *N. benthamiana* leaves using TRIzol reagent (Invitrogen, Waltham, MA, USA, Cat#15596026), and cDNA was generated using the GoScript reverse transcription system (Promega, Madison, WI, USA, Cat#A5001). The target genes were amplified from cDNA with PrimeSTAR^®^ GXL DNA Polymerase (Takara, Kusatsu, Shiga, Japan, Cat#R050A) and cloned into pQBV3 by homologous recombination (Vazyme, Cat#C115), and then recombined into pDEST-VYNE/VYC or pEarleyGate103 by Gateway^®^ Technology (Invitrogen, Cat#12535). The primers are listed in Supplementary Table S1.

### 2.2. Agrobacterium-Mediated Transient Expression

*Agrobacterium tumefaciens* GV3101 strains carrying recombinant binary vectors were used to infiltrate *N. benthamiana* leaves. Agrobacteria were grown in LB medium containing appropriate antibiotics at 28 °C under agitation overnight.

The bacterial cells were collected by centrifugation at 1800× *g* and resuspended in infiltration buffer (10 mM MgCl_2_, 10 mM MES, pH 5.6, and 200 µM acetosyringone [AS]). The suspension was adjusted to the optimal OD_600_ for each specific assay (typically 0.5–0.6; see figure legends for BiFC-specific adjustments). For TurboID labeling, exogenous biotin was dissolved in the same infiltration buffer to a final concentration of 200 µM and infiltrated into *N. benthamiana* leaves. TMV-GFP (TRBO: a high-efficiency tobacco mosaic virus RNA-based overexpression vector) and SMV-GFP [26] were used as infectious clones for resistance verification. GFP fluorescence was detected by long-wave (365 nm) UV at 3–5 days post-infiltration (dpi).

### 2.3. TurboID Sample Preparation

*N. benthamiana* leaves infiltrated with agrobacteria harboring TurboID constructs were prepared at 42 h post infiltration (hpi), to which 200 μM biotin solution was supplemented at 36 h. Four leaves from each plant were then harvested and immediately frozen in liquid nitrogen. Three biological replicates were performed and total protein was extracted with a protein lysis buffer containing protease inhibitor (Solarbio, Beijing, China, Cat#BC3720). After vortex mixing, the samples were placed on ice for 30 min and centrifuged at 4 °C and 16,500× *g* for 10 min. The supernatant was collected and aliquoted and packaged for western blot assay and protein quantification by BCA protein assay (Takara, Cat#T9300A). The rest of the supernatant was run through the Zeba™ Spin Desalting Column (Thermo Scientific, Waltham, MA, USA, Cat#89893) to remove the excess biotin in lysates, and then mixed with streptavidin-coated magnetic beads (Invitrogen, Cat#65001) for biotinylated proteins enrichment. Washing and capturing with magnets were performed as the literature described [14], and the beads were finally dissolved in 1 mL 50 mM ammonium bicarbonate (pH 8.0) for western blot and LC-MS/MS analysis.

### 2.4. Protein Digestion, TMT Labeling, and MS Analysis

After enrichment and purification, the protein magnetic beads samples were treated with DTT to a final concentration of 10 mM. The samples were heated to 65 °C and incubated for 15 min. Then, 0.5 µg Trypsin (Promega, Cat#V5117) and 0.1 µg LysC (FUJIFILM Wako, Osaka, Japan, Cat#125-05061) were added and incubated overnight at 37 °C. Afterwards, 0.5 µg Trypsin and 0.05 µg LysC were added to the sample again, and incubated at 37 °C for 3 h. The sample was then added with iodoacetamide to a final concentration of 2.5 mM and reacted at 37 °C in the dark for 30 min. Subsequently, formic acid was added to adjust the pH of the solution to about 2–3. The magnetic beads were separated using a magnetic rack and the supernatant was recovered. The supernatant samples containing peptide segments were desalinated using SOLA^TM^ SPE 96-well plates (Thermo Scientific, Cat#60209). The eluted peptide segments were dried using a vacuum concentrator and dissolved in water. Peptide quantification was performed using Nanodrop 1000 (Thermo Scientific). After quantification, the samples were labeled with TMT isotope labels (Thermo Scientific, Cat#90111), and then an equal amount of labeled samples were mixed for reverse phase chromatography separation and mass spectrometry loading. The data were analyzed by Proteome Discoverer 2.4 software (Thermo Scientific), and the database was the *N. benthamiana* proteome file downloaded from the Solanaceae Genomics website (SOL Genomics, https://solgenomics.net/organism/Nicotiana_benthamiana/genome, accessed on 15 June 2022).

### 2.5. Western Blot Analysis

Extracted or enriched proteins were mixed with SDS-PAGE loading buffer (CWBIO, Taizhou, China, Cat#CW0027S), boiled at 95–100 °C for 10 min, and centrifuged briefly at 12,000× *g* for 1 min before loading. SDS-PAGE gels were prepared at room temperature, and vertical electrophoresis was performed according to the procedures described [27]. Proteins were then transferred to a 0.45 μm PVDF membrane (BioSharp, Hefei, China, Cat#IPVH00010) by Trans-Blot Semi-Dry Transfer Cell (Bio-Rad, Hercules, CA, USA, Cat#1703940). For HA-tagged proteins, the membrane was blocked with 5% fat-free milk in TBST for 1 h at room temperature, then incubated with the HA primary antibody (Sigma-Aldrich, St. Louis, MO, USA, Cat#H3663) diluted 1:1000 in TBST at 4 °C overnight, followed by incubation with the corresponding secondary antibody (TransGen Biotech, Beijing, China, Cat#HS201-01) diluted 1:1000 in TBST for 1 h at room temperature. For biotinylated proteins, the membrane was blocked with 2.5% BSA in TBST for 1.5 h at room temperature, then incubated with Streptavidin-HRP antibodies (Abcam, Cambridge, UK, Cat#ab7403) diluted 1:120,000 in TBST for 1 h at room temperature. Protein bands were visualized using SuperSignal^®^ West Pico Plus Chemiluminescent Substrate (Thermo Scientific, Cat#NCI5080) according to the manufacturer’s instructions, and the Bio-Rad ChemiDoc XRS+ chemiluminescent imager was used for exposure imaging.

### 2.6. VIGS Assay

The 300 bp region of the candidate gene was selected based on the SGN VIGS Tool (http://vigs.solgenomics.net), and the synthesized cDNA fragments (GenScript) were cloned into TRV-RNA2 vector pYL156 (pTRV2), which was linearized with *Xba*I/*Kpn*I. *Agrobacterium* cultures containing pTRV1 vector and recombinant pTRV2 vector were mixed at a 1:1 proportion, adjusted to a final OD_600_ of 0.5–0.6, and infiltrated into 4-week-old transgenic SRC7-containing *N. benthamiana* leaves. After 10 days compared with TRV-PDS infiltrated plants, the systemic leaves were infiltrated with TMV-GFP or SMV-GFP infectious clones for resistance verification, and GFP fluorescence was observed by long-wave (365 nm) UV light at 5 dpi. Three sets of biological replicates were conducted for each candidate gene. The sequences of these VIGS fragments are provided in Supplementary Table S2.

### 2.7. Reverse Transcription-Quantitative PCR (RT-qPCR) Analysis

Total RNA extraction and reverse transcription were performed as described above, and the reverse-transcription quantitative PCR (RT-qPCR) using gene-specific primers was carried out in an Analytikjena qTOWER^3^ qPCR instrument (Analytik jena, Jena, Germany) using TransStart^®^ Tip Green qPCR SuperMix (TransGen Biotech, Cat#AQ141). The data were normalized to *N. benthamiana ACTIN* expression by the cycle threshold (C_t_) 2^−∆∆Ct^ method according to the literature [28], and analyzed by Graphpad Prism 7.0 software (GraphPad Software, San Diego, CA, USA). All experiments were repeated at least three times. Primers used in this study are listed in Supplementary Table S1.

### 2.8. Confocal Microscopy

Live leaf-tissue samples were cut from *N. benthamiana* leaves infiltrated with agrobacteria at 36–48 hpi, and observed under a Zeiss LSM710 confocal laser scanning microscope (CLSM) (Zeiss, Jena, Germany). Imaging parameters were set using Zeiss ZEN 2011 Black Version software according to the manufacturer’s instructions, and the excitation wavelengths were set to 488 nm and 514 nm for GFP and Venus fluorescence, respectively. Images within a panel were acquired using the same confocal settings and processed uniformly with Zeiss ZEN Blue 3.2.0 Version software.

### 2.9. Yeast Two-Hybrid Assay

The target gene was cloned into Y2H vectors pGADT7 or pGBKT7 using homologous recombination (Vazyme, Cat#C115). After sequencing verification, the recombinants were co-transformed into yeast strain Y2HGold. The preparation of competent yeast cells and plasmid transformation procedures followed the protocol in the Yeastmaker^TM^ user manual (Clontech, Mountain View, CA, USA, Cat#PT1172-1). The transformed yeast suspension was spread on yeast-nutrient-deficient solid culture medium, with additional X-α-Gal, and incubated at 30 °C. The colony verification and photography were performed after 4–6 days.

## 3. Results

### 3.1. Identification of SRC7^TN^-Proximal Candidate Proteins in N. benthamiana Using TurboID Proximity Labeling

SRC7 is an atypical TNX protein from *G. max* containing TIR, NBS, and a C-terminal basic secretory protein (BSP) domain. It exhibits broad-spectrum antiviral activity against multiple plant viruses including SMV, PPV, PVY, and TMV. We previously demonstrated that the SRC7^TN^ confers full resistance to SMV and TMV in *N. benthamiana*, indicating that this region is sufficient to trigger immune responses [25].

Previous work also demonstrated that antiviral resistance mediated by SRC7^TN^ strictly depends on its nuclear localization; forced cytoplasmic retention via an added nuclear export signal abolishes resistance, whereas NLS point-mutations disrupt both nuclear accumulation and antiviral activity [25]. GFP-tagged SRC7^TN^ was directly visualized in the nucleus in that study [25].

**Figure 1:**
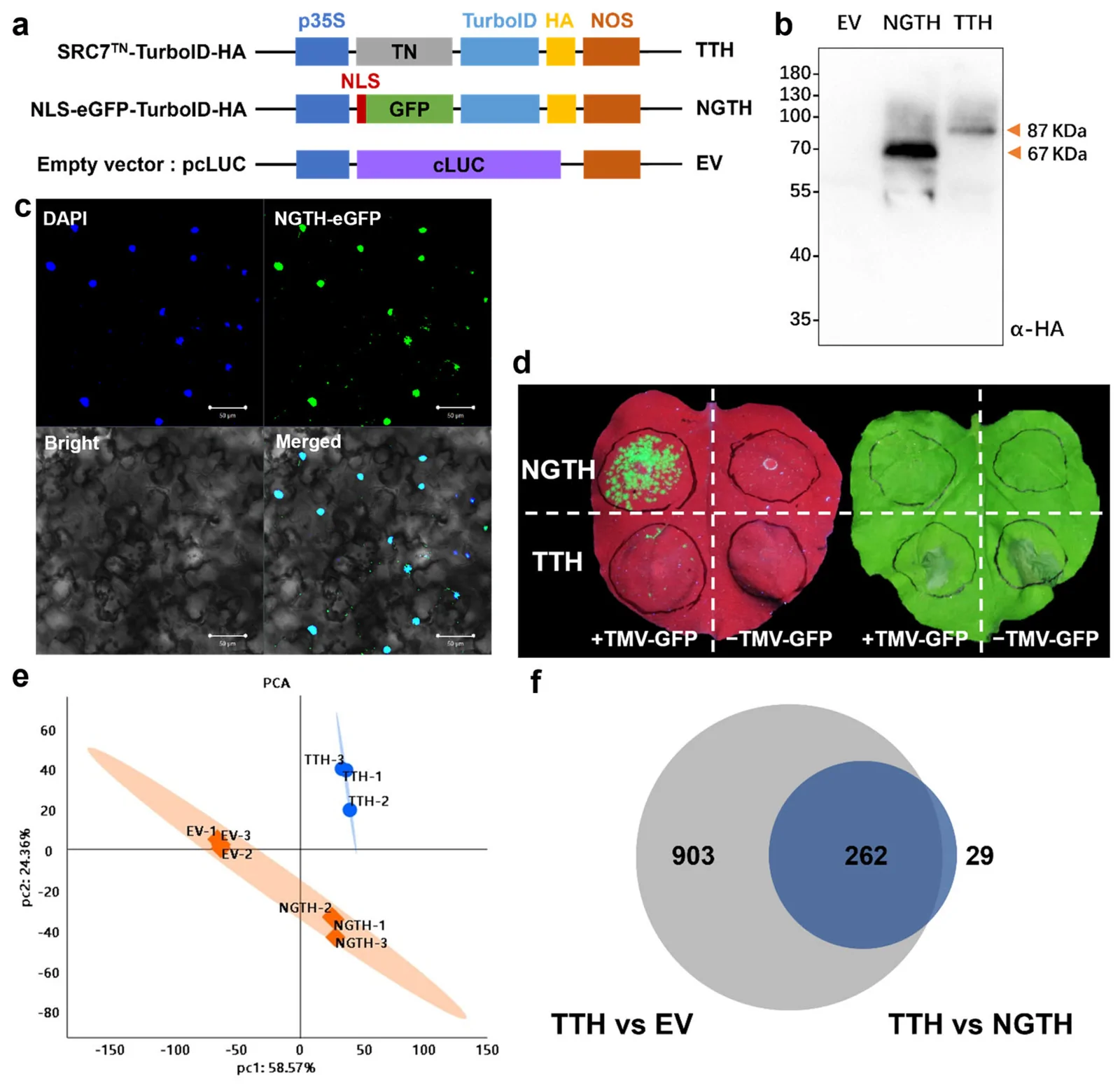
Construction and functional verification of the SRC7^TN^-TurboID system for screening proximal candidate proteins. (**a**) Structural diagrams of three experimental vectors. (**b**) Immunoblot assay verified the normal expression of NGTH and TTH proteins in *N. benthamiana*. (**c**) Subcellular localization observation of NGTH fusion protein. Note: TTH has no fluorescent tag; evidence for its nuclear import is indirect [25], scale bar = 50 µm. (**d**) Antiviral phenotype detection of TTH and NGTH after TMV-GFP infection. Two parallel biological replicate leaves are shown: left, bright-field image; right, UV image. For each leaf: upper regions infiltrated with NGTH, lower regions with TTH; left side +TMV-GFP, right side −TMV-GFP. HR cell death only appears in a subset of infiltrated regions. (**e**) Principal component analysis revealed distinct grouping and high intra-group consistency of proteomic samples (*n* = 3). (**f**) Venn analysis screened overlapping enriched proteins from TTH vs. EV and TTH vs. NGTH groups.

Given that *N. benthamiana* is a well-established model species for studying plant-pathogen interactions and R protein-mediated immunity, we sought to identify SRC7^TN^-proximal candidate proteins in this system using the recently developed TurboID-based PL technique. To this end, we generated the construct pCAMBIA1300-SRC7^TN^-TurboID-HA, in which TurboID and a hemagglutinin (HA) tag were fused in-frame to the C-terminus of SRC7^TN^. TurboID enables biotinylation-based capture of proximal proteins, while the HA tag facilitates detection of the recombinant protein. As a negative control, we generated pCAMBIA1300-NLS-GFP-TurboID-HA, in which a nuclear localization signal (NLS) was fused to the N-terminus of GFP-TurboID-HA to direct nuclear localization, matching the subcellular localization of SRC7^TN^ and allowing us to control for non-specific nuclear proteins labeled by TurboID (Figure 1a).

These constructs were introduced into *A. tumefaciens* and transiently expressed in *N. benthamiana* leaves. At 3 dpi, western blot analysis confirmed the expression of SRC7^TN^-TurboID-HA and NLS-GFP-TurboID-HA proteins at the expected molecular weights (Figure 1b). Only the NGTH construct carries a GFP fluorescent reporter and can be directly visualized by confocal microscopy. Subcellular localization assays confirmed nuclear accumulation of NGTH (Figure 1c). Notably, TTH lacks a fluorescent reporter and cannot be imaged directly by confocal microscopy. Importantly, fusion of TurboID-HA did not compromise the antiviral activity of SRC7^TN^ against TMV infection, indicating that the recombinant SRC7^TN^-TurboID-HA retains its biological function and is suitable for subsequent of proximal candidate proteins. Given that nuclear entry is an absolute prerequisite for SRC7^TN^-triggered antiviral immunity [25], the retained antiviral phenotype provides indirect evidence that the SRC7^TN^-TurboID-HA fusion protein is capable of nuclear import. The bright-field and UV images in Figure 1d are from parallel biological replicate leaves with identical treatments (Figure 1d).

The experiment included three groups with three biological replicates each. (1) EV (empty vector) group: plants infiltrated with a cLUC empty vector construct with biotin treatment, serving as the empty-backbone control. (2) NGTH group: plants expressing NLS-GFP-TurboID-HA with biotin treatment to control for non-specific nuclear interactions. (3) TTH group: plants expressing SRC7^TN^-TurboID-HA with biotin treatment as the test group for identifying SRC7^TN^-associated proteins.

At 3 dpi, biotin was infiltrated into leaves expressing the recombinant proteins, followed by incubation at room temperature for 6 h. Leaves were harvested and total proteins were extracted. Efficient and consistent biotinylation was confirmed by streptavidin-HRP immunoblotting across all biological replicates (Supplementary Figure S1; quantitative densitometry data in Table S3). Free biotin was removed using desalting columns, and biotinylated proteins were enriched with streptavidin beads. Following on-bead trypsin digestion, approximately 50 μg of peptides was recovered from each sample. Peptides were labeled with isobaric tandem mass tag (TMT) reagents and analyzed using two-dimensional liquid chromatography–tandem mass spectrometry (2D LC-MS/MS), followed by quantitative analysis of affinity-purified peptides.

In total, 4644 proteins with TMT intensity > 0 were identified across 9 samples. A high-confidence dataset was generated using strict filtering criteria. The filtered set included 3573 proteins, accounting for 76.9% of all identified proteins. Principal component analysis (PCA) revealed clear separation among the three experimental groups and strong reproducibility within each group of three replicates (Figure 1e). Proteomic data were further supported by correlation analysis (Supplementary Figure S2). Global protein expression distribution and data normalization were further verified (Supplementary Figure S3).

SRC7^TN^-proximal candidate proteins were defined as proteins with a false discovery rate (FDR)-corrected q-value < 0.05 and > 1.5-fold enrichment in TTH relative to both NGTH and EV. According to these criteria, 1165 differentially enriched proteins were identified in TTH vs. EV, and 291 differentially enriched proteins in TTH vs. NGTH (Supplementary Figure S4). Venn diagram analysis identified 262 overlapping proteins between the two datasets, which were designated as high-confidence SRC7^TN^-proximal candidate proteins (Figure 1f). The term “high-confidence” refers to candidates filtered by the statistical thresholds defined in this study, rather than direct physical interactions. Detailed information including accession numbers, annotations, and quantitative proteomic data for these 262 proteins is provided in Supplementary Table S4. GO enrichment analyses were performed separately on the full lists of enriched proteins from the TTH vs. EV and TTH vs. NGTH comparisons to characterize their global functional profiles (Supplementary Figure S5). Functional categories related to plant immunity, protein modification and nuclear regulation were enriched in both datasets.

### 3.2. Functional Analysis of SRC7^TN^-Proximal Candidate Proteins via Overexpression Assay

To validate whether the TurboID-identified proteins participate in SRC7-mediated resistance to SMV and TMV, twenty-one key proteins were carefully selected from the 262 candidate proteins for further analysis (Table 1).

From the 262 high-confidence SRC7^TN^-proximal candidates, 21 candidates were selected for functional validation. Proteins from the filtered 97-protein subset (removing NGTH vs. EV background proteins) were prioritized, together with consideration of enrichment magnitude, plant immunity-related GO annotation and subcellular-localization information. Published reports describing their roles in plant immune or antiviral responses were also considered as an important criterion during candidate selection. Among these 21 candidates, four proteins (*RIN4*, *PRPX1*, *PRPX2* and *DRB4*) were prioritized mainly based on previously documented immune-related functions, and these four proteins were not included in the 97-protein subset.

**Figure 2:**
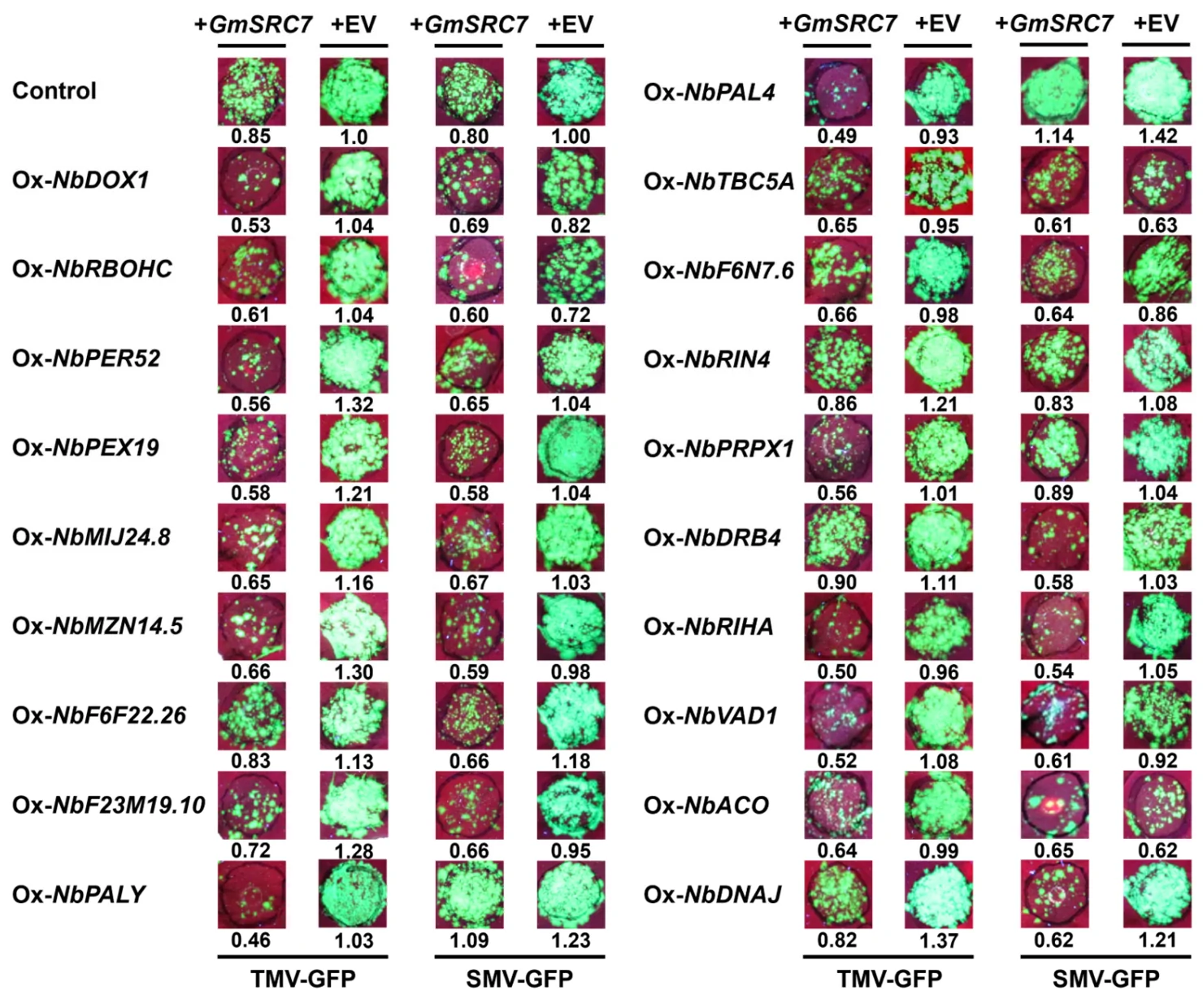
Phenotypic identification of candidate proteins regulating antiviral resistance. Representative leaf fluorescence images of TMV-GFP and SMV-GFP infection. Each candidate gene was expressed alone or co-expressed with *SRC7*, and viral accumulation was evaluated through GFP fluorescence intensity. Relative fluorescence values were quantified for intuitive comparison of viral proliferation levels.

Considering the high similarity of flanking sequences within two paralogous gene pairs (*PAL2*/*PAL4* and *PRPX1*/*PRPX2*), these paralogs could not be amplified individually. One amplicon was recovered from each pair, and sequencing confirmed the obtained clones were *PAL4* and *PRPX1*, respectively. Full-length cDNA of the resulting 19 genes encoding these key proteins were successfully cloned and used for subsequent functional analyses. To characterize the roles of these genes in antiviral immunity, transient overexpression assays were conducted in *N. benthamiana*. Each candidate gene was expressed with the empty vector (EV) or co-expressed with *SRC7*; EV was supplemented to normalize total plasmid amount for agroinfiltration, followed by inoculation with GFP-labeled SMV or TMV to monitor viral proliferation and antiviral activity (Figure 2).

RT-qPCR analysis was performed to detect the transcript abundance of the nineteen target genes under both TMV and SMV infection conditions, either in the presence or absence of SRC7. The results demonstrated that all target genes were efficiently overexpressed, with transcript levels significantly higher than those in the EV control group (***, *p* < 0.001) (Supplementary Figure S6a–d). Viral fluorescence intensity was quantified using ImageJ 1.53t (National Institutes of Health, Bethesda, MD, USA) and was normalized to the average signal of the EV control (Supplementary Figure S7a–d). Combined analyses of gene expression and viral phenotypes revealed that the regulatory effects of the nineteen genes on SRC7-mediated immunity were highly virus-specific.

Eight genes, namely *NbPER52*, *NbPEX19*, *NbMIJ24.8*, *NbMZN14.5*, *NbF23M19.10*, *NbTBC5A*, *NbRIHA*, and *NbDNAJ*, functioned as the regulators that significantly enhanced SRC7-mediated resistance against both SMV and TMV. These genes exhibited prominent transcriptional upregulation upon SRC7 co-expression under both viral infections, accompanied by markedly reduced GFP fluorescence indicative of restricted viral spread (Figure 2, Supplementary Figure S7a–d). Three additional genes, *NbDOX1*, *NbRBOHC*, and *NbACO*, displayed clear TMV-specific antiviral activity. Their expression was strongly induced in the presence of TMV but showed little change or a slight reduction under SMV infection, consistent with the observation that these genes contributed to resistance against TMV but not SMV (Figure 2, Supplementary Figure S7a–d).

Among the SMV-specific regulatory genes, *NbDRB4* significantly enhanced SRC7-triggered resistance to SMV, whereas *NbPALY* and *NbPAL4* remarkably attenuated this resistance. Notably, *NbPALY* alone exerted antiviral effects against TMV, and its expression pattern under SMV infection was fully consistent with the corresponding viral infection phenotype (Figure 2, Supplementary Figure S7a–d). The remaining five genes, including *NbF6N7.6*, *NbF6F22.26*, *NbVAD1*, *NbRIN4*, and *NbPRPX1*, showed weak or negligible effects on viral proliferation, with no obvious differences in expression between SRC7 co-infiltrated and non-co-infiltrated groups (Figure 2, Supplementary Figure S7a–d).

Collectively, the expression levels of core antiviral genes were tightly correlated with their inhibitory effects on viral movement, while virus-specific and weakly responsive genes exhibited distinct expression profiles matching their functional outcomes. These findings illustrate that SRC7 mediates antiviral immunity by differentially regulating downstream immune-related genes in a virus-dependent manner.

### 3.3. Functional Analysis of SRC7^TN^-Proximal Candidate Proteins via TRV-Based VIGS

To further investigate the roles of SRC7-interacting proteins in SRC7-mediated immunity, we employed a TRV-based VIGS system [29,30] to systemically silence these genes in *N. benthamiana* plants overexpressing *SRC7* (*OxSRC7Nb*). The candidate genes were subjected to VIGS to comprehensively assess their functional roles in SRC7-mediated resistance, followed by inoculation with GFP-labeled SMV or TMV to monitor viral proliferation and antiviral activity.

**Figure 3:**
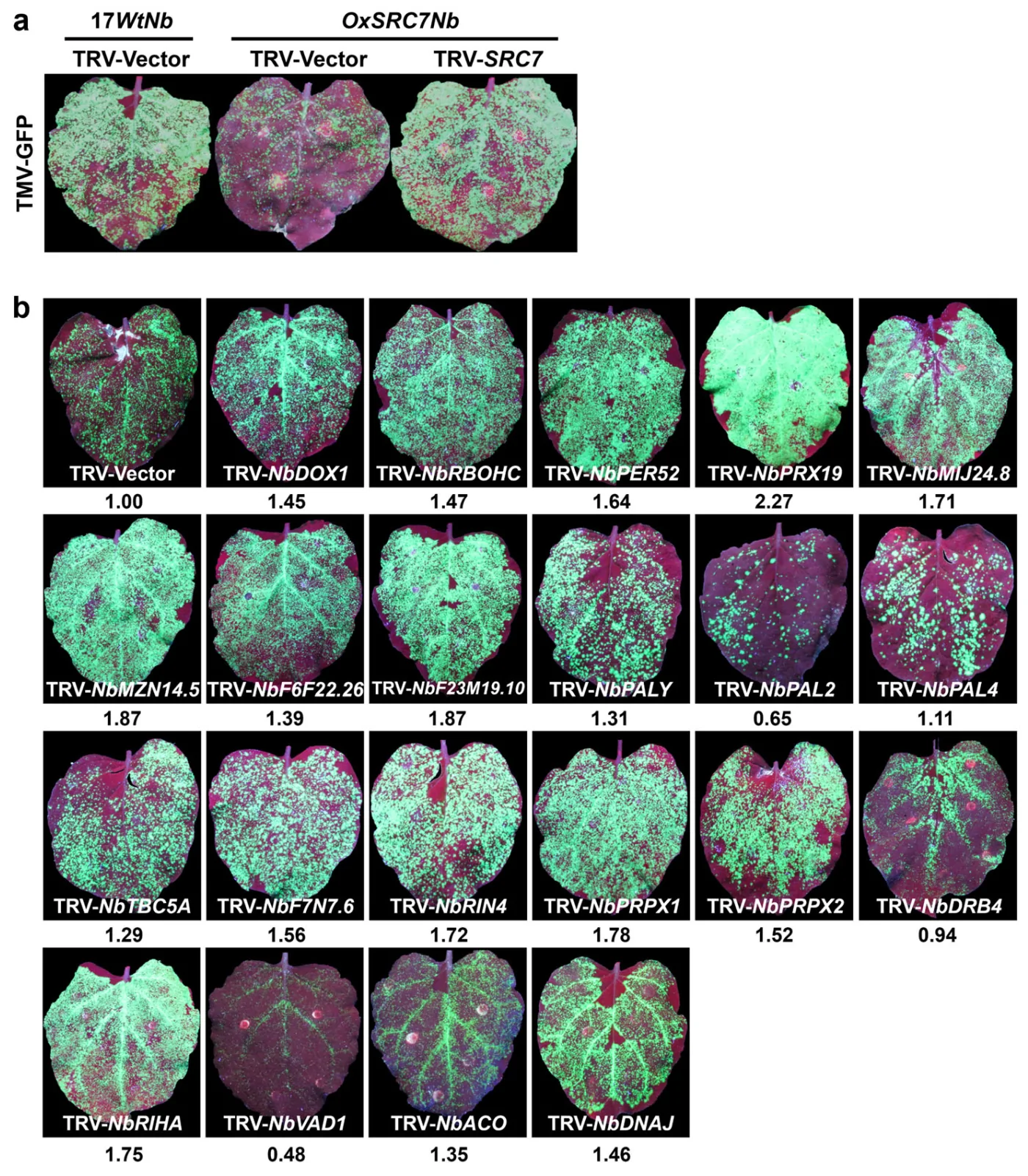
Functional screening of candidate genes regulating the antiviral response mediated by *SRC7*. (**a**) Phenotypic validation of the antiviral effect of *SRC7* overexpression against TMV-GFP. Infection phenotypes were compared among different plant genotypes under different treatments. (**b**) TMV-GFP infection phenotypes after silencing of individual candidate genes via VIGS. The relative viral accumulation level, indicated by GFP fluorescence intensity, is shown below each leaf. Silencing of different genes led to varying effects on the antiviral response.

**Figure 4:**
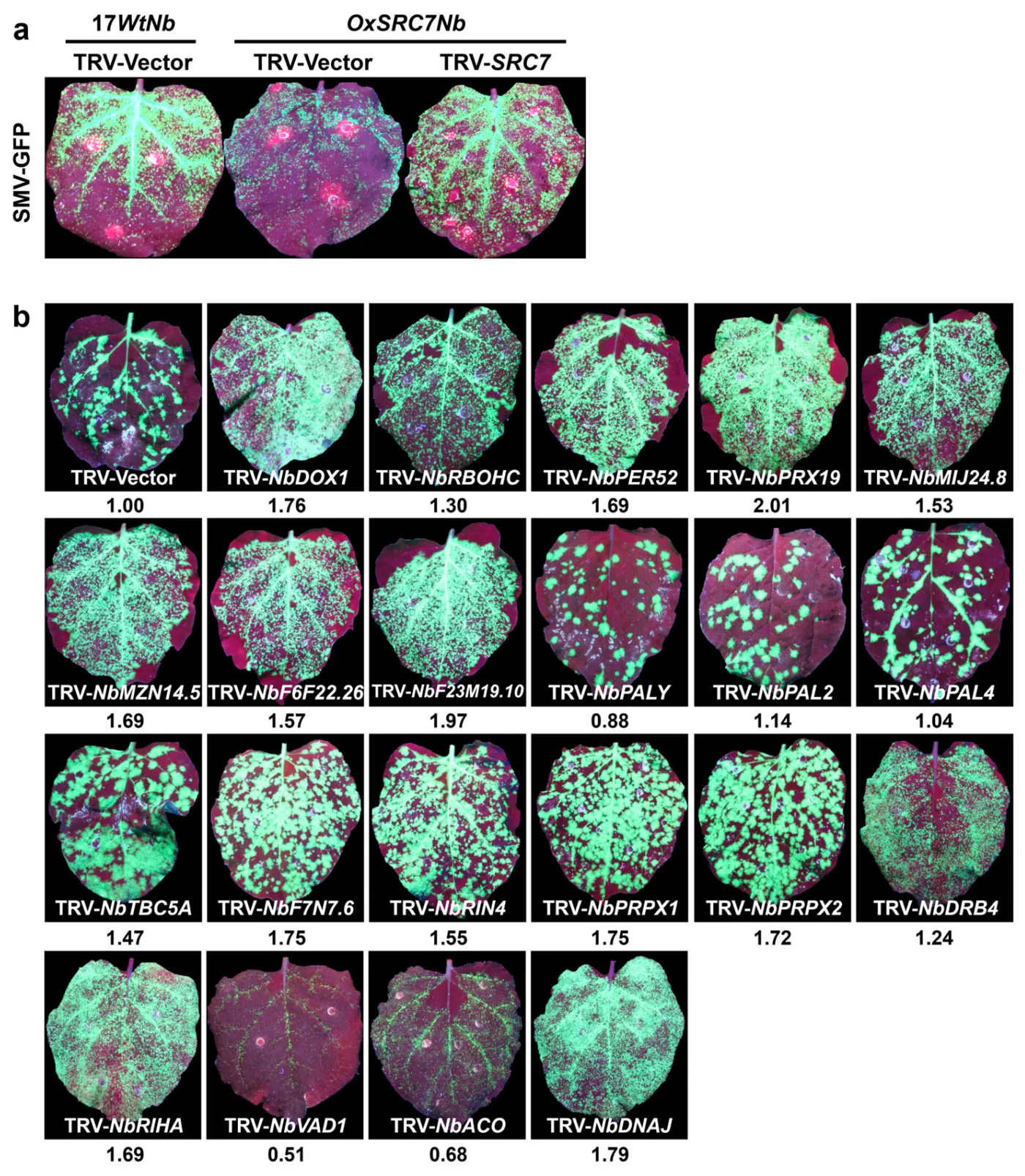
Functional screening of candidate genes regulating the antiviral response mediated by *SRC7* against SMV. (**a**) Phenotypic validation of the antiviral effect of *SRC7* overexpression against SMV-GFP. Infection phenotypes were compared among different plant genotypes under different treatments. (**b**) SMV-GFP infection phenotypes after silencing of individual candidate genes via VIGS. The relative viral accumulation level, indicated by GFP fluorescence intensity, is shown below each leaf. Silencing of different genes led to varying effects on the antiviral response.

Viral phenotypes in the control groups were consistent with previous findings (Figure 3a and Figure 4a). Wild-type *N. benthamiana* (*17WtNb*) plants and *OxSRC7Nb* plants treated with the empty TRV vector (TRV-Vector) served as negative and positive controls, respectively. *OxSRC7Nb* plants displayed robust resistance to both TMV-GFP and SMV-GFP, characterized by markedly reduced GFP fluorescence and localized necrosis. In contrast, *17WtNb* plants and *OxSRC7Nb* plants treated with TRV targeting endogenous *SRC7* (TRV-*SRC7*) exhibited strong systemic green fluorescence, confirming that silencing of *SRC7* completely abolished host resistance. Qualitative viral phenotypes of all control groups are shown in Figure 3a and Figure 4a. Quantitative analysis of viral GFP fluorescence intensity for VIGS-silenced plants, using TRV-Vector (EV) as the internal reference control, is presented in Supplementary Figure S8a,b.

Subsequent VIGS analyses revealed that silencing of most candidate genes significantly compromised SRC7-mediated resistance (Figure 3b and Figure 4b). In the TMV-GFP treatment, silencing of 17 genes (*NbDOX1*, *NbRBOHC*, *NbPER52*, *NbPEX19*, *NbMIJ24.8*, *NbMZN14.5*, *NbF6F22.26*, *NbF23M19.10*, *NbPALY*, *NbTBC5A*, *NbF6N7.6*, *NbRIN4*, *NbPRPX1*, *NbPRPX2*, *NbRIHA*, *NbACO*, and *NbDNAJ*) significantly impaired resistance compared with the TRV-Vector control, resulting in increased viral GFP intensity (Supplementary Figure S8a). Similarly, in the SMV-GFP treatment, silencing of 16 genes (*NbDOX1*, *NbRBOHC*, *NbPER52*, *NbPEX19*, *NbMIJ24.8*, *NbMZN14.5*, *NbF6F22.26*, *NbF23M19.10*, *NbTBC5A*, *NbF6N7.6*, *NbRIN4*, *NbPRPX1*, *NbPRPX2*, *NbDRB4*, *NbRIHA*, and *NbDNAJ*) significantly enhanced viral accumulation (Supplementary Figure S8b).

Notably, several genes exhibited distinct virus-specific regulatory effects. Silencing of *NbPALY* and *NbACO* specifically reduced resistance to TMV, while silencing of *NbDRB4* specifically impaired resistance to SMV. These effects were only observed in the corresponding viral treatment and were reflected in the quantitative fluorescence data (Supplementary Figure S8a,b). Collectively, these findings demonstrate that the majority of identified genes act as key positive regulators required for broad-spectrum SRC7-mediated antiviral defense, while a small subset functions in a virus-specific manner.

To validate VIGS efficiency, transcript levels of all twenty-one target genes were quantified by RT-qPCR in systemic leaves (Supplementary Figure S9). All genes were significantly downregulated compared with the TRV-Vector control, with relative expression levels ranging from 5% to 76% of the control. Statistical analysis confirmed extremely significant differences (*p* < 0.0001) for all genes except *NbDNAJ* (*p* < 0.002). These data confirm efficient silencing of target genes and indicate that the enhanced viral susceptibility in *OxSRC7Nb* plants resulted from downregulation of these immune-related factors, rather than off-target effects of the VIGS system.

### 3.4. Verification of Eleven SRC7^TN^ Interactors via BiFC Assay

To further confirm the physical interactions between SRC7^TN^ and the candidate interactors identified above, bimolecular fluorescence complementation (BiFC) assays were performed for one-to-one verification. From the candidates identified by TurboID-based screening and functional assay, eleven candidate genes were selected for interaction assay, including *DOX1*, *RBOHC*, *PER52*, *PEX19*, *MZN14.5*, *F23M19.10*, *TBC5A*, *RIHA*, *VAD1*, *ACO* and *DNAJ*. These genes were cloned into the pDEST-GWVYCE vector, and transiently co-expressed with SRC7^TN^, which was cloned into the pDEST-GWVYNE vector, in *N. benthamiana* leaves.

**Figure 5:**
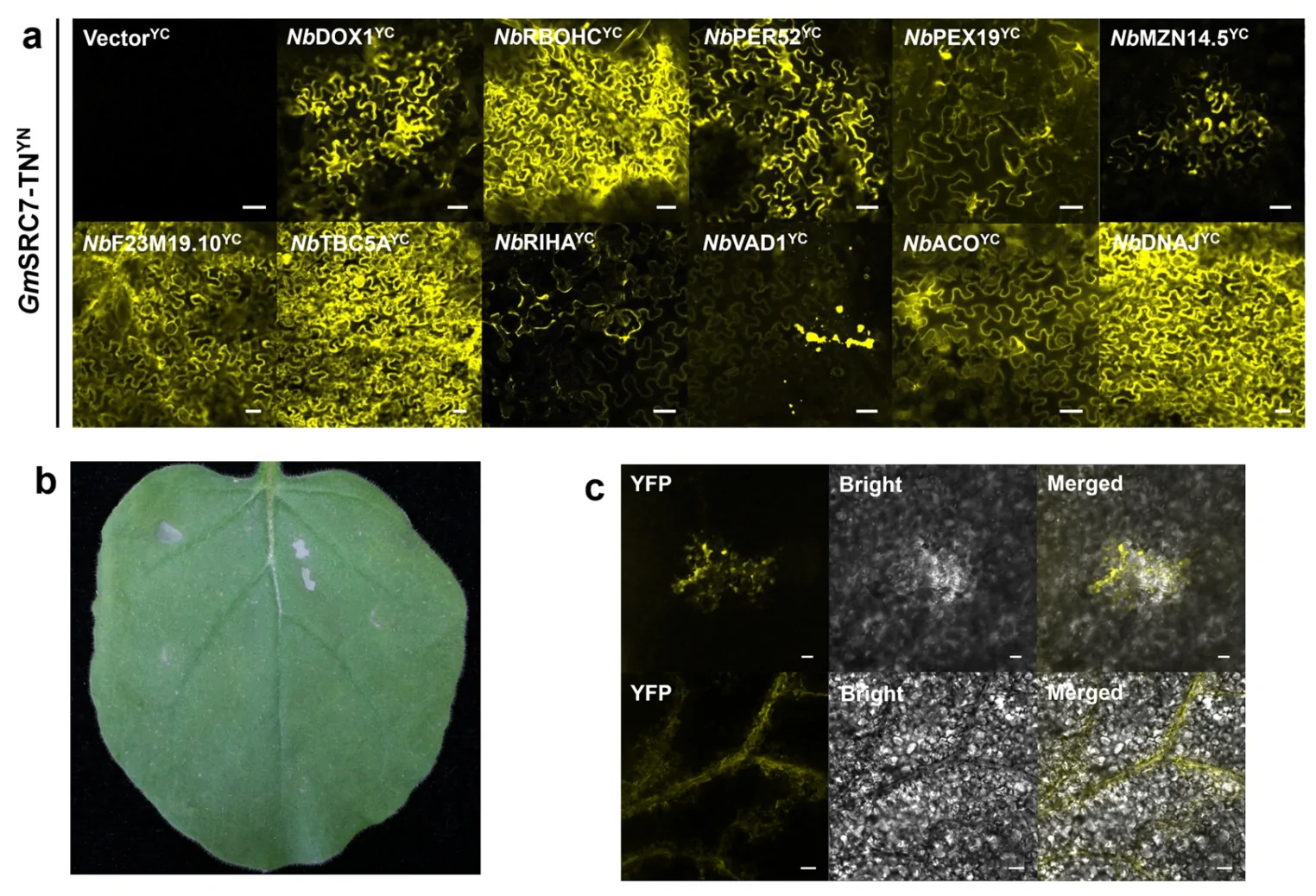
Verification of the interaction between SRC7^TN^ and candidate proteins via BiFC. (**a**) BiFC assay showing the interaction between SRC7^TN^ and candidate proteins. This panel is for qualitative screening of protein–protein interactions and not intended for detailed subcellular localization analysis. YFP fluorescence was observed in leaf epidermal cells co-expressing SRC7^TN^-YN and candidate protein–YC constructs. Scale bars = 50 µm. (**b**) Representative leaf showing the agroinfiltration sites used for the BiFC assay. (**c**) Confocal microscopy of BiFC samples showing cytological phenotypes at 48 h post agroinfiltration. SRC7^TN^ (pDEST-GWVYNE- SRC7^TN^) and interacting candidate proteins (pDEST-GWVYCE-candidate) were transiently co-expressed in wild-type *N. benthamiana*. *A. tumefaciens* GV3101 cultures were infiltrated at a final OD_600_ of 0.4 for SRC7^TN^ and 0.6 for candidate genes. Leaf tissues were imaged at 48 h after infiltration. YFP fluorescence, bright-field and merged channels are shown. Fluorescence signals were observed at the edge of translucent lesion regions and along leaf veins. Scale bars = 50 µm.

Since SRC7^TN^ without the BSP domain easily induces basal resistance and interferes with fluorescence imaging, the final OD_600_ of the *A. tumefaciens* strain GV3101 cultures was adjusted to 0.4 for SRC7^TN^ and 0.6 for the target genes. Laser confocal imaging was conducted at 36–48 h after transient expression. No fluorescence signal was detected under 514 nm excitation when empty vectors (nVenus + cVenus) were used as the negative control. In contrast, yellow fluorescence was observed when SRC7^TN^ was co-expressed with each of the 11 candidate genes, and most signals were distributed along cell contours (Figure 5a). Among these combinations, *DOX1*, *RBOHC*, *PER52*, *PEX19*, *F23M19.10*, *TBC5A*, *ACO* and *DNAJ* showed stronger interaction signals with SRC7^TN^ in *N. benthamiana*.

Notably, under bright-field imaging, cell outlines in the experimental groups were generally indistinct or unevenly translucent, which was not observed in the negative control or non-inoculated *N. benthamiana* leaves. Local punctate translucent necrosis appeared in leaves at 36 h after transient expression (Figure 5b). At 48 h after transient expression, cell structures were further disrupted, and yellow fluorescence was detected at the edges of translucent regions. These cytological features accompany the co-expression of SRC7^TN^ and its interacting candidates, suggesting a potential association between their physical interaction and immune-associated cellular damage. In some fields of view, fluorescence was detected along opaque fine veins (Figure 5c), which raises the possibility that the SRC7^TN^-containing protein complex or related metabolites may contribute to cell-to-cell immune signaling.

### 3.5. Analysis of Two Nucleoside/Nucleotide Hydrolases MZN14.5 and RIHA

Among the eleven functional interactors further validated above, this study focused on *MZN14.5* and *RIHA*, both belonging to the nucleoside/nucleotide hydrolase family. Existing studies have shown that free nucleotides enhance resistance of *A. thaliana* to *Pseudomonas syringae* pv. *maculicola ES4326* [31,32]. However, no reports are currently available regarding the involvement of nucleoside/nucleotide hydrolases in *R* gene-mediated resistance.

**Figure 6:**
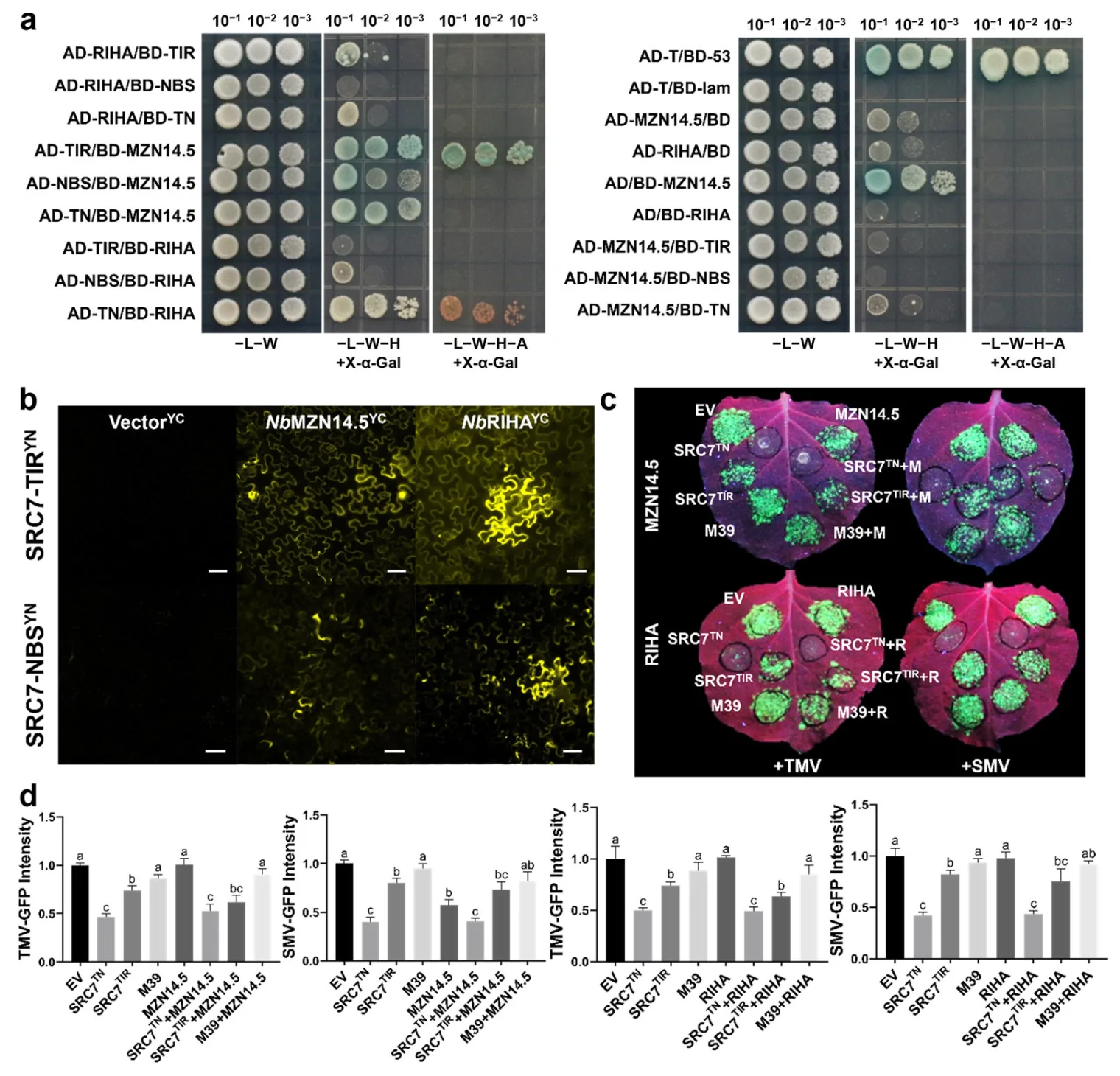
Interaction verification and functional analysis of SRC7^TN^ with candidate proteins. (**a**) Yeast two-hybrid assay of the interactions between SRC7 domains and MZN14.5/RIHA. (**b**) BiFC assay of the interactions between SRC7 TN/TIR/NBS and NbMZN14.5/NbRIHA in planta. Scale bars = 50 µm. (**c**) Representative leaf fluorescence images of TMV-GFP and SMV-GFP infection under different treatments. (**d**) Quantification of relative viral GFP intensity. Data are mean ± SD (*n* = 3). One-way ANOVA followed by Tukey’s multiple comparisons test was performed for statistical analysis. Different letters indicate significant differences (*p* < 0.05).

To clarify the functions of MZN14.5 and RIHA in SRC7-mediated downstream immune responses, their interactions with the TIR and NBS domains of SRC7 were first examined. Y2H assays were performed to investigate the interactions between the two proteins and individual domains of SRC7. The results showed that MZN14.5 interacted with SRC7^TIR^, while RIHA interacted with SRC7^TN^ (Figure 6a). In addition, BiFC assays revealed that co-expression of MZN14.5 or RIHA with SRC7^TIR^ produced distinct fluorescence distributed along the cell contours, whereas co-expression with SRC7^NBS^ resulted only in irregular fluorescence. The Y2H and BiFC results were largely consistent, validating the physical interactions of MZN14.5 and RIHA with the respective domains of SRC7, and also supporting the reliability of TurboID-based PL for screening interacting proteins (Figure 6b).

To further verify whether MZN14.5 and RIHA can substitute the NBS domain of SRC7 as nucleoside/nucleotide hydrolases and activate downstream immune responses by interacting with the TIR domain, viral resistance assays were performed. The SRC7 mutant M39, which loses antiviral activity due to a G20R mutation at position 20 in the αA1 motif of the SRC7^TIR^ domain, was used as a control. MZN14.5 and RIHA were individually co-expressed with SRC7^TIR^ for antiviral analysis. The results showed that, in contrast to the strong resistance conferred by SRC7 and the lost resistance in the M39 group, the viral fluorescence intensity was not significantly reduced in the MZN14.5/RIHA + SRC7 groups at the quantitative level, indicating that MZN14.5 and RIHA could not enhance the resistance of SRC7 against TMV or SMV (Figure 6d). Notably, a slight reduction in viral GFP fluorescence was visually observable in both the SRC7 + target gene groups compared with the groups expressing SRC7 alone (Figure 6c).

Previous studies demonstrated that SRC7 containing only the TIR domain without the NBS domain exhibits significantly decreased antiviral activity. Combined with our results, under our experimental conditions, co-expression of MZN14.5 or RIHA with SRC7^TIR^ did not rescue the impaired antiviral activity of SRC7^TIR^ and failed to restore downstream immune signaling, despite the physical interaction between these two proteins and SRC7, as well as a slight visual reduction in viral fluorescence. We note that we cannot exclude the possibility that insufficient protein accumulation or altered subcellular localization of MZN14.5 or RIHA may contribute to this negative phenotype. These results suggest that the synergistic enhancement of SRC7 resistance by MZN14.5 and RIHA depends on the full-length SRC7, and they highlight the essential role of the NBS domain in SRC7-mediated antiviral immunity. To further elucidate the molecular basis underlying the interactions of MZN14.5 and RIHA with SRC7, AlphaFold3 (AF3)-based structural prediction was performed to model their detailed binding interfaces. In good agreement with our Y2H and BiFC assays, AF3 predictions showed that MZN14.5 stably bound to the SRC7^TN^ homotetramer, while RIHA interacted stably with both the SRC7^TN^ homotetramer and SRC7^TN^ monomer (Supplementary Figure S10). These structural observations support the notion that MZN14.5 and RIHA likely modulate SRC7 activation by targeting the functional interfaces of the SRC7^TN^ homotetramer to regulate its assembly or conformational stability, rather than acting as a direct functional substitute for the NBS domain. Together with our antiviral assays, these results further reinforce that MZN14.5 and RIHA depend on the full-length SRC7^TN^ complex to contribute to antiviral immunity, and highlight the importance of the TN domain as a core regulatory platform for SRC7 activation.

## 4. Discussion

Although native genetic evidence in *G. max* would further strengthen the conclusion, heterologous expression in *N. benthamiana* is a widely accepted and genetically sound approach for the identification of NLR interactomes, especially for methodologically demanding PL techniques. TurboID-based screening relies on in vivo proximal labeling efficiency rather than native mutant backgrounds, and the vast majority of published TurboID applications in plants have been performed in heterologous systems [20,21]. Our functional validations using overexpression, VIGS, and BiFC collectively provide robust in planta genetic evidence supporting the roles of identified interactors in SRC7-mediated immunity. Thus, the heterologous system does not compromise the reliability of the proximal-candidate dataset or the functional conclusions.

In this study, we leveraged the functional autonomy of the SRC7^TN^ domain, previously validated to confer complete resistance, to circumvent the heterologous expression challenges and non-specific labeling associated with full-length SRC7. By directly fusing SRC7^TN^ to TurboID, we effectively reduced the overall molecular weight of the chimeric protein, thereby enhancing transient expression efficiency while preserving biotinylation fidelity. Structural prediction using PSIPRED indicated that the junction regions between SRC7^TN^, TurboID, and the eGFP control adopt unstructured coil conformations. This intrinsic flexibility alleviates steric constraints between functional modules, eliminating the necessity for an artificial linker sequence. The omission of a rigid linker not only streamlines system design but also aligns with observations that flexible tethers improve PL resolution [19].

To further refine the temporal window of labeling, we optimized the transient expression duration to 24–48 h, followed by a 6 h biotin incubation prior to harvest. This schedule mitigates two common pitfalls in *Agrobacterium*-mediated plant expression: insufficient target protein accumulation due to abbreviated expression, and non-specific background labeling resulting from prolonged overexpression or RNA silencing onset [33,34]. The inclusion of an EV negative control enabled stringent downstream filtering. By intersecting differentially enriched proteins from (TTH vs. EV) and (TTH vs. NGTH) comparisons, we defined a high-confidence subset of 262 candidates. This multi-control strategy adheres to established proteomic standards for minimizing false discovery rates [24].

GO enrichment analysis of the high-confidence set of SRC7^TN^-proximal candidate proteins revealed significant overrepresentation of biological processes associated with plant immunity, post-translational modification, and nuclear regulation. These data support a model wherein SRC7^TN^ exerts antiviral surveillance by interfacing with both transcriptional machinery and cytoplasmic signaling cascades. As reported in reference [25], SRC7^TN^ exhibits a bipartite distribution: while prominent nuclear fluorescence was observed, SRC7^TN^ also exhibited distinct extranuclear signals. Rather than contradicting the established requirement for nuclear localization in SRC7-mediated resistance, this finding implies a functional synergy between compartments. Nuclear retention likely governs transcriptional reprogramming of defense genes, whereas cytoplasmic residence may facilitate direct modulation of protein folding, trafficking, or oxidative bursts.

Consistent with the latter hypothesis, proximity enrichment of peroxidases suggests that SRC7^TN^ may actively prime or execute reactive oxygen species (ROS) generation. Given that the spatial and temporal balance of ROS is a hallmark of the HR and a key rheostat coordinating immunity with growth [35], SRC7^TN^ interactions with redox-related proteins may constitute a critical checkpoint. Furthermore, BiFC assays captured irregular fluorescent puncta that were predominantly distributed at the boundaries or interior of collapsed mesophyll tissue. Critically, the emergence and expansion of these signals coincided temporally with the progression of local HR lesions. This dynamic pattern suggests that the interaction between SRC7^TN^ and specific candidates precedes visible cell death and may represent an initiating event in HR execution. These observations reinforce the canonical role of NLR proteins in orchestrating localized cell death to restrict systemic viral spread [36].

Comparative analysis with a previously reported interactome dataset for the N protein (an NLR harboring a TN domain) identified 22 common candidate genes, encoding proteins such as helicases, elongation factors, and phosphatases. Notably, protein phosphatase 2C (PP2C) exhibited robust fold changes in both independent datasets (FC = 4.8 and 3.9, respectively). This convergent identification of PP2C underscores the existence of conserved regulatory nodes and metabolic subnetworks utilized by distinct NLR sensors to potentiate plant immunity. Among these, Niben101Scf04626g00009.1 (alpha-dioxygenase 1, *NbDOX1*), Niben101Scf07345g00016.1 (respiratory burst oxidase homolog C, *NbRBOHC*), Niben101Scf02349g03002.1 (peroxidase 52, *NbPER52*), and Niben101Scf00317g06018.1 (peroxisomal biogenesis factor 19, *NbPEX19*) are directly involved in peroxidase-mediated redox reactions. Previous studies have shown that R protein-triggered cell death in plants is closely associated with intracellular ROS bursts [37]. Quantitative analysis showed that DOX1 and PER52 had log_2_ fold change (FC) > 2.0 in TTH vs. EV, while RBOHC and PEX19 exhibited higher log_2_FC in TTH vs. NGTH than in TTH vs. EV.

Niben101Scf07465g02005.1 (TLDc domain-containing nucleolar protein, *NbMIJ24.8*) and Niben101Scf07058g04002.1 (P-loop containing nucleoside triphosphate hydrolase, *NbMZN14.5*) also participate in intracellular redox regulation. The TLDc domain of MIJ24.8 reduces intracellular ROS levels [38], and its *A. thaliana* homolog MZN14.5 responds to singlet oxygen signals under stress conditions [39]. In both TTH vs. EV and TTH vs. NGTH comparisons, MIJ24.8 showed log_2_FC of approximately 2.3, while MZN14.5 showed log_2_FC of approximately 4.7.

Niben101Scf02816g01009.1 (regulator of Vps4 activity in the MVB pathway, *NbF6F22.26*) and Niben101Scf00941g02004.1 (regulator of Vps4 activity in the MVB pathway, *NbF23M19.10*) are involved in vacuolar protein sorting regulation. In plant immunity, endosomal trafficking is closely linked to autophagy, and R protein-triggered HR involves vacuole-mediated autophagy-related processes [40]. Both proteins exhibited log_2_FC > 2.0 in TTH vs. EV and TTH vs. NGTH comparisons.

Niben101Scf04090g02003.1 (phenylalanine ammonia-lyase, *NbPALY*), Niben101Scf12881g00010.1 (*NbPAL2*), and Niben101Scf05442g03015.1 (*NbPAL4*) are involved in SA biosynthesis. Previous studies have confirmed that *R* gene-mediated immune responses are associated with the SA signaling pathway [41], and all three proteins showed log_2_FC > 2.5 in both comparisons.

Niben101Scf04393g00002.1 (TBC1 domain family member 5 homolog A, *NbTBC5A*) and Niben101Scf02112g00006.1 (RabGAP/TBC domain-containing protein, *NbF6N7.6*) belong to the Rab GTPase-activating protein (RabGAP) family. Plant RabGAPs mediate intracellular viral movement; for example, *Nb*RabGAP1 regulates intracellular movement of bamboo mosaic virus (BaMV) [42].

Niben101Scf08799g00001.1 (RPM1-interacting protein 4 transcript 2, *NbRIN4*), Niben101Scf11110g00023.1 (pathogenesis-related protein X1, *NbPRPX1*), Niben101Scf11110g00018.1 (*NbPRPX2*), and Niben101Scf05841g01021.1 (double-stranded RNA-binding protein 4, *NbDRB4*) were not among the common differential proteins after excluding NGTH vs. EV. However, they showed log_2_FC > 2.0 in TTH vs. NGTH and > 4.5 in TTH vs. EV. RIN4 is a key protein for *R* gene-mediated avirulence recognition; in *A. thaliana*, RPM1 and RPS2 require RIN4 to recognize AvrRpm1/AvrRpt2 and activate downstream immune responses [43,44]. PRPX1 and PRPX2 are nuclear membrane proteins that facilitate nuclear protein transport [45] and may be involved in SRC7 nuclear translocation. DRB4 participates in RNA silencing-mediated antiviral defense and interacts with viral silencing suppressors (VSRs) [46].

Niben101Scf03674g00005.1 (pyrimidine-specific ribonucleoside hydrolase RihA, *NbRIHA*) is a pyrimidine-preferring nucleoside hydrolase, and its conserved domain enhances bacterial virulence [47]. Its log_2_FC reached 3.9 in TTH vs. NGTH and 5.5 in TTH vs. EV.

Niben101Scf05173g02010.1 (vascular-associated death 1, *NbVAD1*) and Niben101Scf02918g00003.1 (1-aminocyclopropane-1-carboxylate oxidase, *NbACO*) are associated with plant cell death. VAD1 is involved in HR-like responses, and VAD1-mediated vascular death depends on ethylene and SA pathways [48]. As the final rate-limiting enzyme in ethylene biosynthesis, ACO promotes ethylene production [49] and induces necrotic lesion formation on tobacco leaves upon viral infection [50].

Niben101Scf03817g16010.1 (chaperone protein DnaJ, *NbDNAJ*) is essential for proper protein folding and participates in various stress responses [51]. Quantitative analysis showed that DNAJ exhibited log_2_FC of 2.6 in TTH vs. NGTH and 2.1 in TTH vs. EV.

It should be noted that TurboID captures proteins with spatial proximity to SRC7^TN^ rather than exclusively direct physical interactors. In addition, Y2H and BiFC assays detect protein association in heterologous expression systems and may not fully represent physiological interactions in native plant cells. While this TurboID-based screen has illuminated the proximal interactome of SRC7^TN^, certain limitations warrant acknowledgment. First, the transient expression system employed here cannot fully recapitulate the stoichiometry or compartmental dynamics of endogenous SRC7 within soybean cells. Consequently, the precise molecular mechanisms governing extranuclear interactors remain to be resolved. Orthogonal validation using co-immunoprecipitation (Co-IP) or pull-down assays will be essential to distinguish direct binding partners from constituents of higher-order complexes. Second, the functional consequences of SRC7^TN^ engagement with conserved regulators, particularly PP2C, require detailed dissection to map the downstream signaling cascades that culminate in antiviral immunity. Future investigations will prioritize three directions: (1) functional characterization of priority candidates to construct a comprehensive immune signaling network centered on SRC7^TN^; (2) assays using diverse viral pathosystems to delineate the breadth and specificity of SRC7-mediated defense; and (3) integration of soybean protoplast or stable transgenic systems to validate the physiological relevance of identified interactions, thereby providing a robust foundation for molecular breeding strategies aimed at enhancing soybean antiviral resilience.

By optimizing the TurboID PL methodology for the functional SRC7^TN^ domain, we have delineated a high-confidence interactome that bridges nuclear and cytoplasmic immune activities. Our findings establish that SRC7^TN^ engages in multifaceted processes, including protein modification and oxidative burst, through spatially coordinated interactions. Moreover, the identification of shared partners with other NLR immune receptors points toward conserved regulatory architecture in plant innate immunity. This study provides a valuable conceptual framework for deciphering NLR function in soybeans and delivers a suite of candidate genes and technical resources to advance antiviral molecular improvement.

## 5. Conclusions

In summary, this study successfully employed TurboID-mediated PL to establish a high-confidence dataset of high-confidence SRC7^TN^-proximal candidate proteins of the functional SRC7 TN domain in *N. benthamiana*, identifying 262 candidate host proteins potentially involved in SRC7-triggered antiviral immunity. Through systematic overexpression and TRV-based VIGS functional screening, combined with BiFC verification, we confirmed multiple previously unreported SRC7^TN^-proximal regulatory proteins that positively modulate plant broad-spectrum antiviral resistance via redox homeostasis, protein trafficking, SA signaling, and stress-responsive protein folding. Several candidates exhibited distinct virus-specific regulatory effects against TMV and SMV, revealing a sophisticated and multilayered regulatory network underlying NLR-mediated antiviral defense.

Collectively, our findings demonstrate that the SRC7 TN domain serves as a core signaling hub that integrates nuclear and cytoplasmic immune responses through dynamic protein–protein interactions. This work expands the mechanistic understanding of atypical TIR-NBS-BSP NLR function in antiviral immunity and provides a valuable resource of host candidate genes for future molecular dissection of plant immune pathways. Furthermore, the optimized TurboID screening strategy established in this study offers a reliable technical reference for exploring weak and transient NLR protein interactions in plant systems, laying a foundation for the future improvement of crop antiviral resistance through molecular breeding.

## Figures and Tables

**Table 1:** Candidate genes selected for functional validation.

No.	Gene ID	Name	Annotation	TTH vs. EV		TTH vs. NGTH	
				log_2_FC	*p*-Value	log_2_FC	*p*-Value
1	Niben101Scf04626g00009.1	*Nb*DOX1	Alpha-dioxygenase 1	3.68	0.0095	2.64	0.0156
2	Niben101Scf07345g00016.1	*Nb*RBOHC	Respiratory burst oxidase homolog protein C	1.76	0.0028	2.26	0.0021
3	Niben101Scf02349g03002.1	*Nb*PER52	Peroxidase 52	2.33	0.0033	1.95	0.0040
4	Niben101Scf00317g06018.1	*Nb*PEX19	Peroxisomal biogenesis factor 19	1.63	0.0119	1.69	0.0131
5	Niben101Scf07465g02005.1	*Nb*MIJ24.8	TLDc domain-containing nucleolar protein	2.32	0.0008	2.31	0.0007
6	Niben101Scf07058g04002.1	*Nb*MZN14.5	P-loop containing nucleoside triphosphate hydrolases	4.74	0.0024	4.77	0.0025
7	Niben101Scf02816g01009.1	*Nb*F6F22.26	Regulator of Vps4 activity in the MVB pathway	2.70	0.0029	2.07	0.0050
8	Niben101Scf00941g02004.1	*Nb*F23M19.10	Regulator of Vps4 activity in the MVB pathway	2.84	0.0000	2.01	0.0001
9	Niben101Scf04090g02003.1	*Nb*PALY	Phenylalanine ammonia-lyase	4.56	0.0092	3.06	0.0154
10	Niben101Scf12881g00010.1	*Nb*PAL2	Phenylalanine ammonia-lyase 2	3.82	0.0002	2.64	0.0003
11	Niben101Scf05442g03015.1	*Nb*PAL4	Phenylalanine ammonia-lyase 4	3.21	0.0008	2.55	0.0001
12	Niben101Scf04393g00002.1	*Nb*TBC5A	TBC1 domain family member 5 homolog A	2.92	0.0001	2.14	0.0005
13	Niben101Scf02112g00006.1	*Nb*F6N7.6	RabGAP/TBC domain-containing protein	1.83	0.0001	1.61	0.0007
14	Niben101Scf08799g00001.1	*Nb*RIN4	RPM1 interacting protein 4 transcript 2	4.72	0.0000	2.63	0.0001
15	Niben101Scf11110g00023.1	*Nb*PRPX1	Pathogenesis-related protein X1	7.00	0.0000	2.29	0.0001
16	Niben101Scf11110g00018.1	*Nb*PRPX2	Pathogenesis-related protein X2	10.82	0.0017	2.16	0.0161
17	Niben101Scf05841g01021.1	*Nb*DRB4	Double-stranded RNA-binding protein 4	6.47	0.0000	2.01	0.0001
18	Niben101Scf03674g00005.1	*Nb*RIHA	Pyrimidine-specific ribonucleoside hydrolase RihA	5.55	0.0003	3.95	0.0005
19	Niben101Scf05173g02010.1	*Nb*VAD1	Vascular-associated death 1	2.93	0.0019	2.03	0.0059
20	Niben101Scf02918g00003.1	*Nb*ACO	1-aminocyclopropane-1-carboxylate oxidase	2.60	0.0001	1.77	0.0013
21	Niben101Scf03817g16010.1	*Nb*DNAJ	Chaperone protein DnaJ	2.06	0.0027	2.59	0.0013

Notes: log_2_FC, log_2_ fold change; *p*-value, FDR-corrected *q*-value. TTH, SRC7^TN^-TurboID-HA; EV, empty vector; NGTH, NLS-GFP-TurboID-HA.

## Data Availability

The datasets generated and analyzed during the current study are available from the corresponding author upon reasonable request.

## Supplementary Materials

The following supporting information can be downloaded at https://www.mdpi.com/article/10.3390/biology15211949/s1, Figure S1: Detection of in vivo biotinylation efficiency of TurboID fusion proteins; Figure S2: Repeatability assessment of proteomic data via correlation analysis; Figure S3: Overall quality control of proteomic quantitative data; Figure S4: Volcano plot showing protein enrichment in the TTH vs. EV and TTH vs. NGTH comparisons; Figure S5: Gene Ontology functional enrichment analysis of proximal candidate proteins; Figure S6: RT-qPCR analysis of candidate gene expression under different treatments; Figure S7: Quantification of viral GFP fluorescence intensity under different treatment combinations; Figure S8: Quantification of viral accumulation following VIGS-mediated silencing of candidate genes; Figure S9: Validation of VIGS-mediated gene silencing efficiency; Figure S10: Structural modeling of interactions between *GmSRC7*^TN^ and its candidate interactors using AlphaFold3; Table S1: The PCR primer sequences used in this study; Table S2: VIGS silencing fragment sequences used in this study; Table S3: Densitometry quantification results for the streptavidin-HRP immunoblot (Figure S1); Table S4: List of 262 high-confidence candidate proximal proteins of GmSRC7^TN^ identified by TurboID-based proximity labeling and quantitative proteomics.
