# Supplementary material for: TurboID Screening and Functional Analysis of the Interactome of the Broad-Spectrum Antiviral Protein Soybean Resistance Cluster 7 (SRC7)
Source: Biology (Basel). 2026 Oct 29;15(21):1949. doi: 10.3390/biology15211949 (PMC13761510; doi:10.3390/biology15211949)
Supplement: Supplementary file 1 [file biology-15-01949-s001.zip › Supplementary_Figures_and_Legends.pdf]

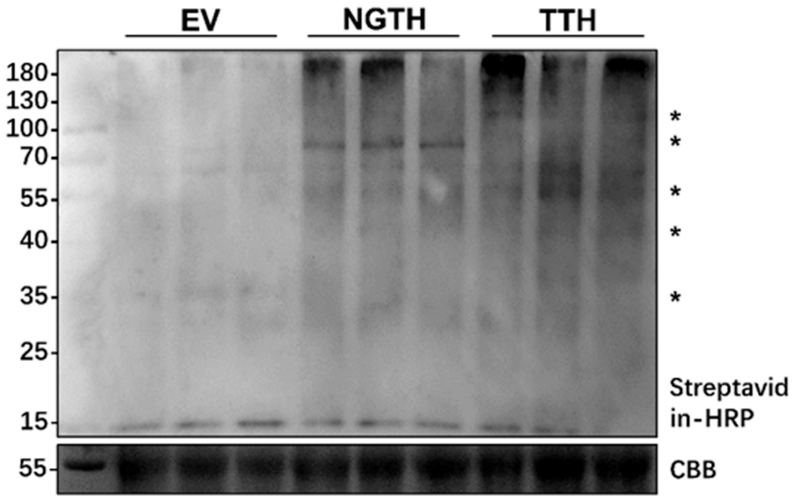

Figure S1 Detection of in vivo biotinylation efficiency of TurboID fusion proteins. Streptavidin-HRP immunoblotting was used to detect biotinylated proteins in EV, NGTH and TTH groups. Specific enrichment signals were observed in TurboID-expressing samples. Coomassie brilliant blue (CBB) staining was used as the total protein loading control to ensure consistent sample loading. Densitometric quantification of band intensity ratios is provided in Table S4.

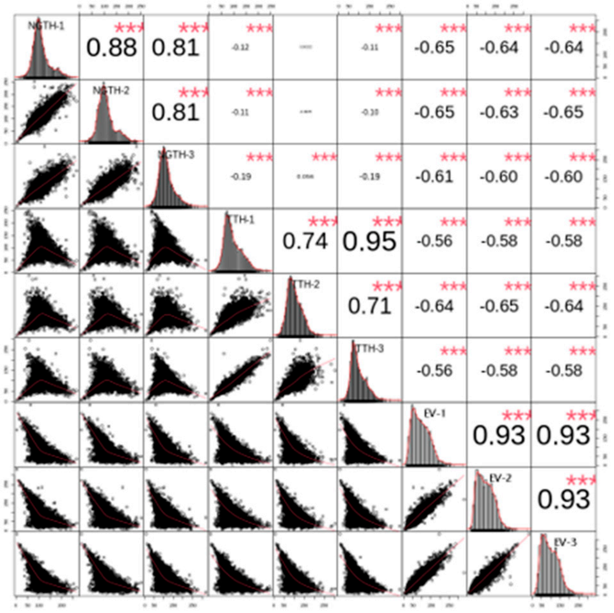

Figure S2 Repeatability assessment of proteomic data via correlation analysis. Comprehensive pairwise correlation analysis was performed across all biological replicates. Protein abundance distributions, pairwise correlation coefficients and sample scatter plots are presented. High correlation coefficients ($R > 0.70$, ***$p < 0.001$) confirmed good experimental reproducibility.

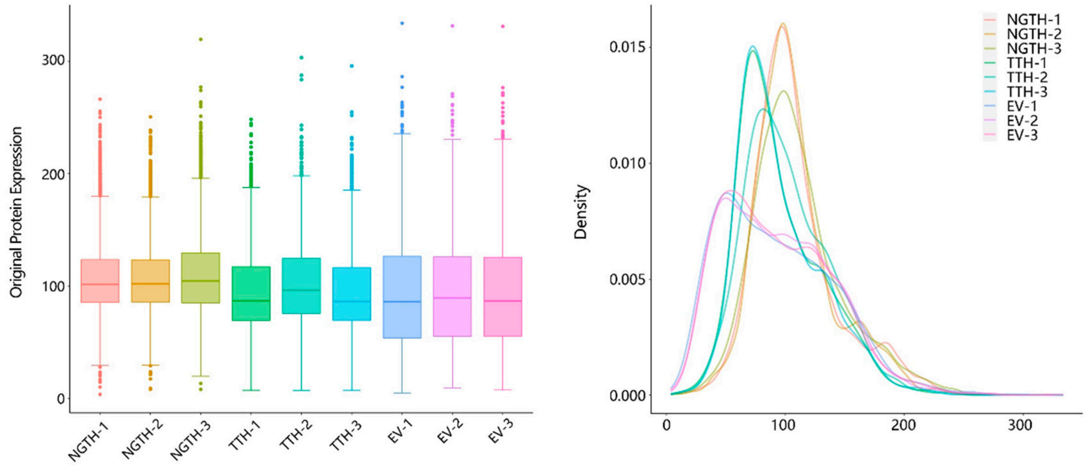

Figure S3 Overall quality control of proteomic quantitative data. Box plots and density plots intuitively displayed the overall distribution characteristics of protein abundance in all samples. The consistent data distribution among groups proved that normalization was reliable and the dataset was suitable for subsequent differential analysis.

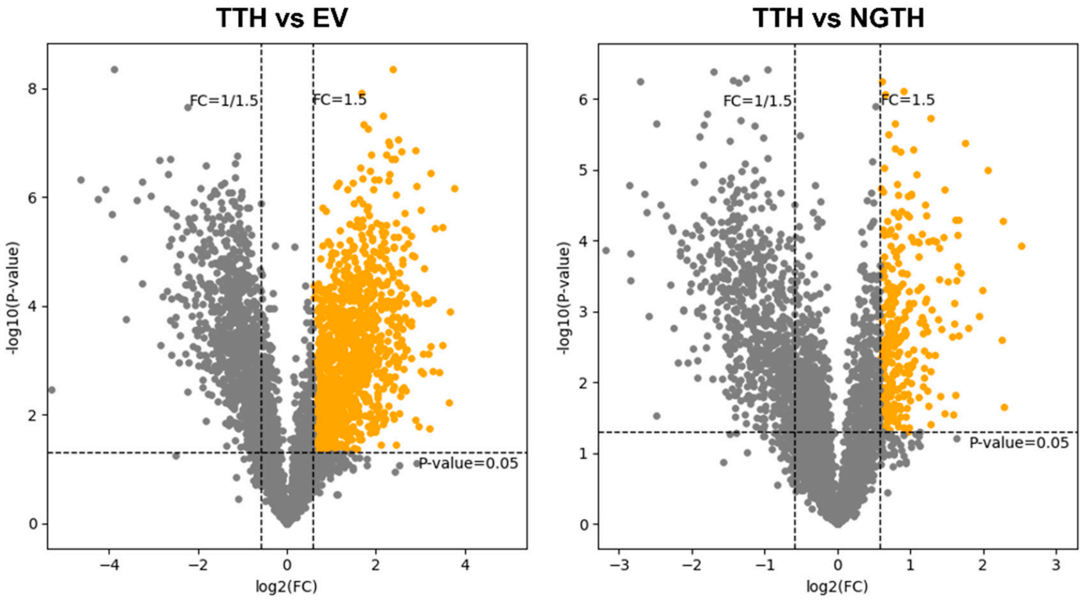

Figure S4 Volcano plot showing protein enrichment in the TTH vs. EV and TTH vs. NGTH comparisons. The horizontal axis represents $\log_2$ fold change ($\log_2$FC). The vertical axis displays $-\log_{10}$-transformed raw *P*-values for overall data distribution visualization. Protein screening was performed using FDR-corrected *q*-value < 0.05 and FC > 1.5, rather than the raw *P*-values shown in this plot. Orange dots mark proteins meeting our enrichment criteria.

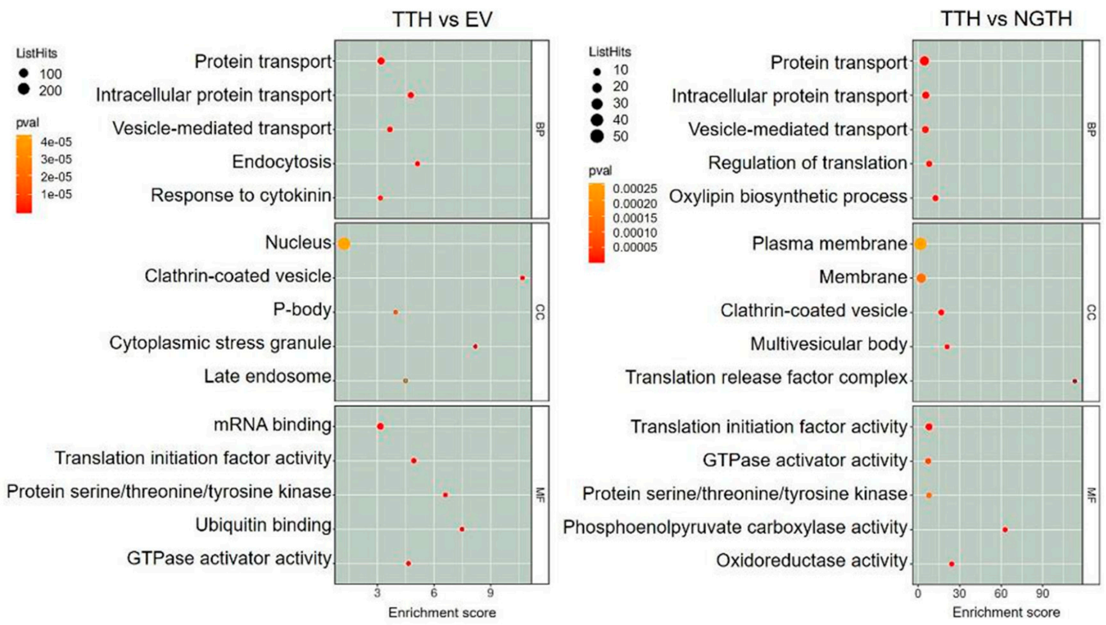

Figure S5 Gene Ontology functional enrichment analysis of candidate interacting proteins. Bubble plots summarized the enrichment results of biological process, cellular component and molecular function. Bubble size corresponds to the number of enriched proteins, and color gradient indicates the significance of $-\log_{10}(p)$ values, reflecting the core functional classification of candidate proteins.

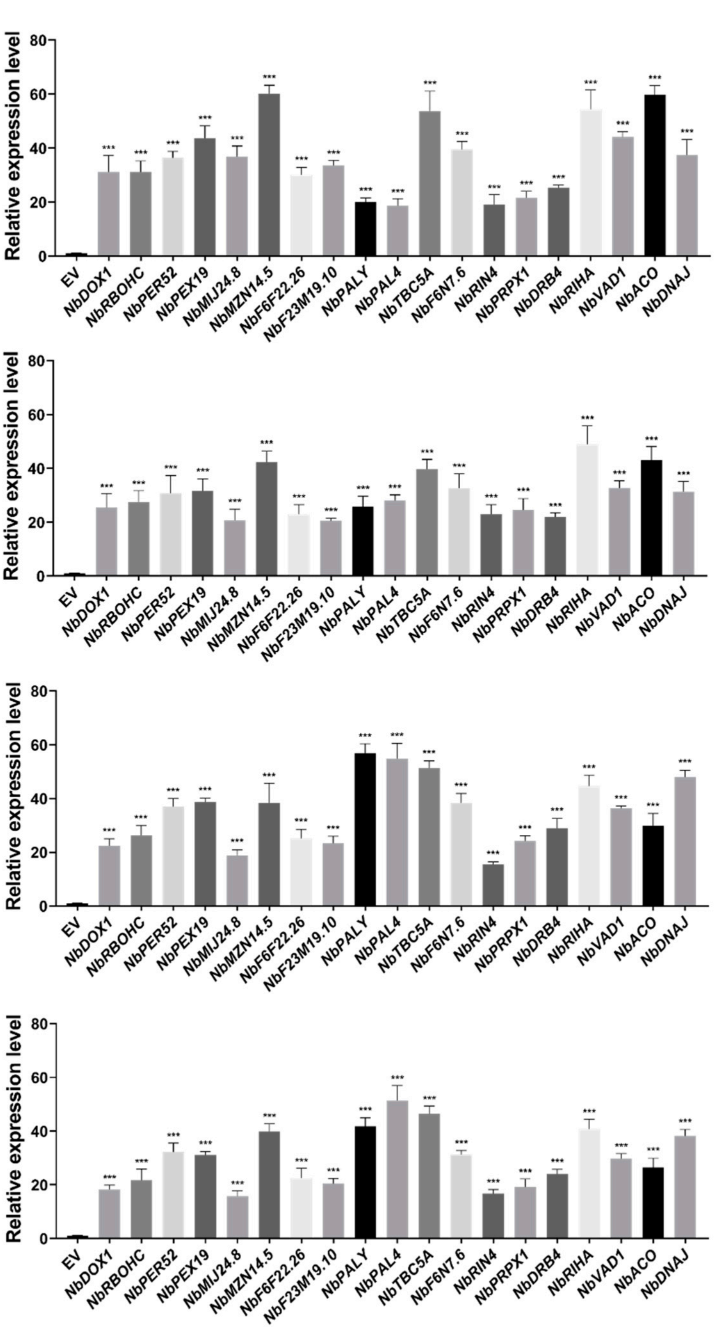

Figure S6 RT-qPCR analysis of candidate gene expression under different treatments. The transcription levels of candidate genes were quantified by RT-qPCR. Candidate genes were expressed with EV or co-expressed with *GmSRC7*; EV was supplemented to normalize total plasmid amount for agroinfiltration. Transcript levels were normalized to the reference gene *NbActin*, and calculated relative to the EV control (EV expression level set as 1). Treatments: a candidate gene + *GmSRC7* + TMV, b candidate gene + TMV, c candidate gene + *GmSRC7* + SMV, d candidate gene + SMV. Data are mean ± SD (n = 3). Asterisks denote significant differences vs. EV control (***$P < 0.001$, one-way ANOVA with Dunnett's test).

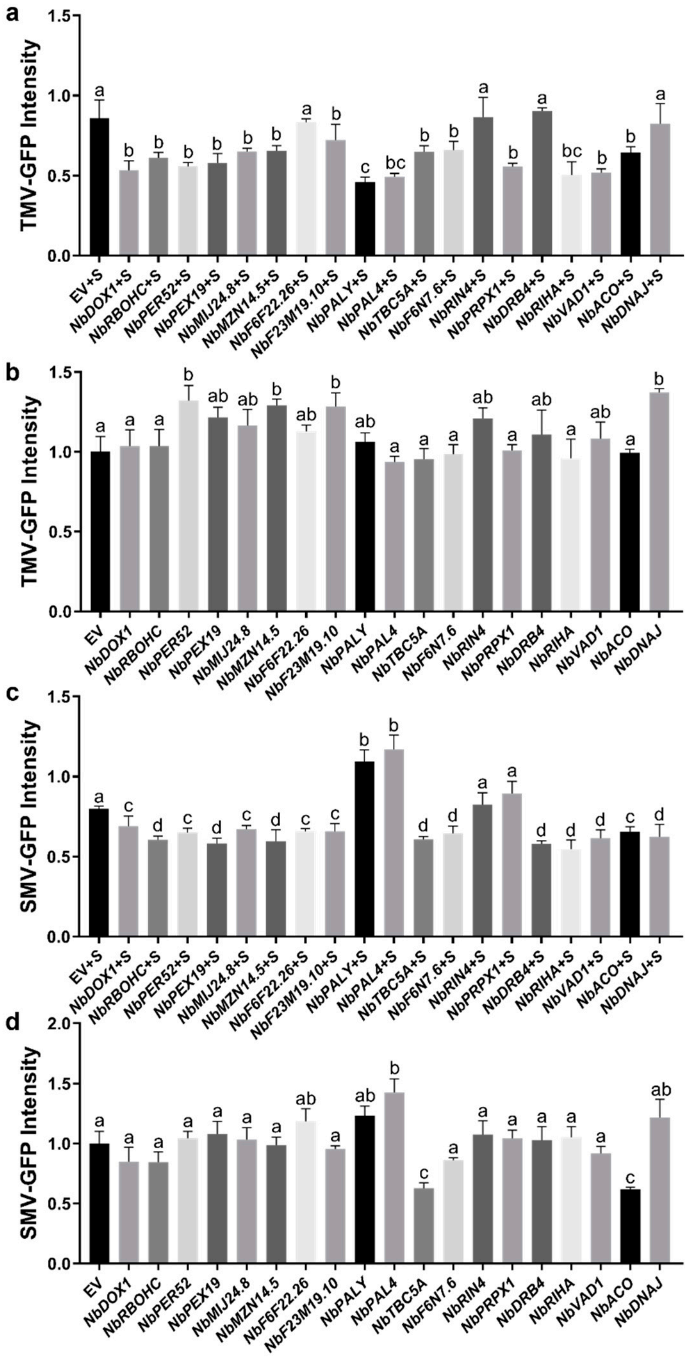

Figure S7 Quantification of viral GFP fluorescence intensity under different treatment combinations. GFP fluorescence intensity was measured to assess TMV-GFP (a, b) and SMV-GFP (c, d) accumulation. Candidate genes were expressed with EV or co-expressed with *GmSRC7*; EV was supplemented to normalize total plasmid amount for agroinfiltration. Viral fluorescence intensity was normalized to the EV control (EV signal level set as 1). Fluorescence signals were quantified using ImageJ software. Treatments: a, c candidate gene + *GmSRC7* + virus; b, d candidate gene + virus. Data are presented as mean ± SD ($n = 3$). Different lowercase letters indicate significant differences among groups ($P < 0.05$, one-way ANOVA followed by Tukey's multiple range test).

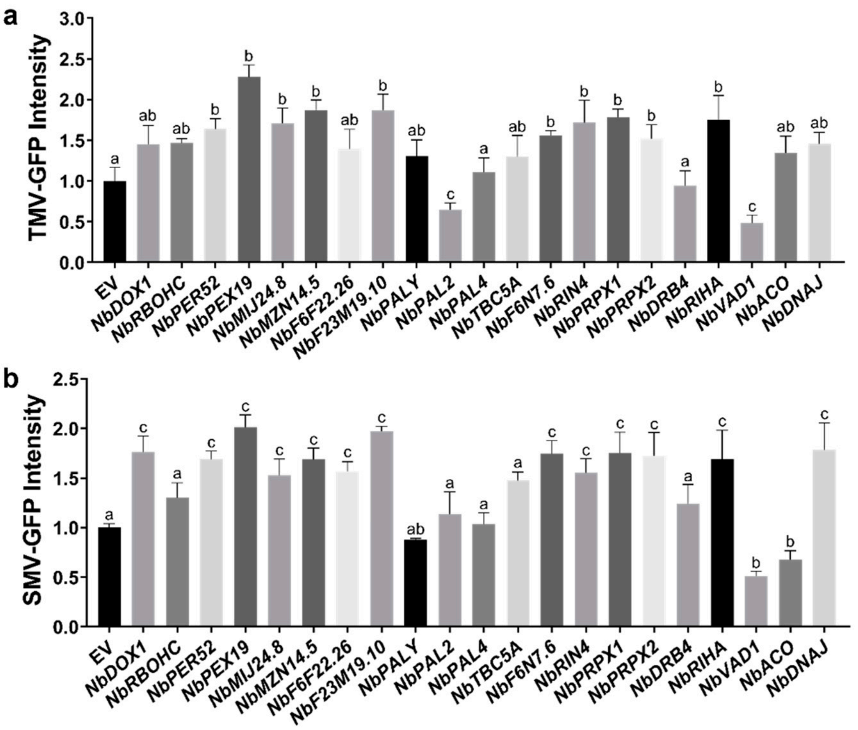

Figure S8 Quantification of viral accumulation following VIGS-mediated silencing of candidate genes. a TMV-GFP fluorescence intensity in plants with VIGS-silenced candidate genes. b SMV-GFP fluorescence intensity in plants with VIGS-silenced candidate genes. GFP fluorescence intensity was measured to assess viral accumulation. TRV-Vector (EV) was used as the internal reference control for VIGS treatments. Qualitative phenotypes of additional control groups are shown in Figure 3a and Figure 4a. Data are presented as mean ± SD (n = 3). Different lowercase letters indicate significant differences among groups (P < 0.05, one-way ANOVA followed by Tukey's multiple range test).

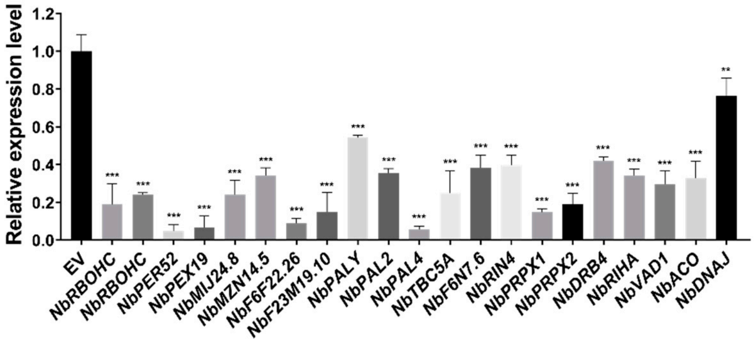

Figure S9 Validation of VIGS-mediated gene silencing efficiency. RT-qPCR was performed to detect the transcript levels of candidate genes in plants where each gene was individually silenced via VIGS. The relative expression level in the EV control was set to 1. All data are presented as mean ± SD (*n* = 3). Asterisks indicate significant differences compared with the EV control (****P* < 0.001, ***P* < 0.01, one-way ANOVA followed by Dunnett's test).

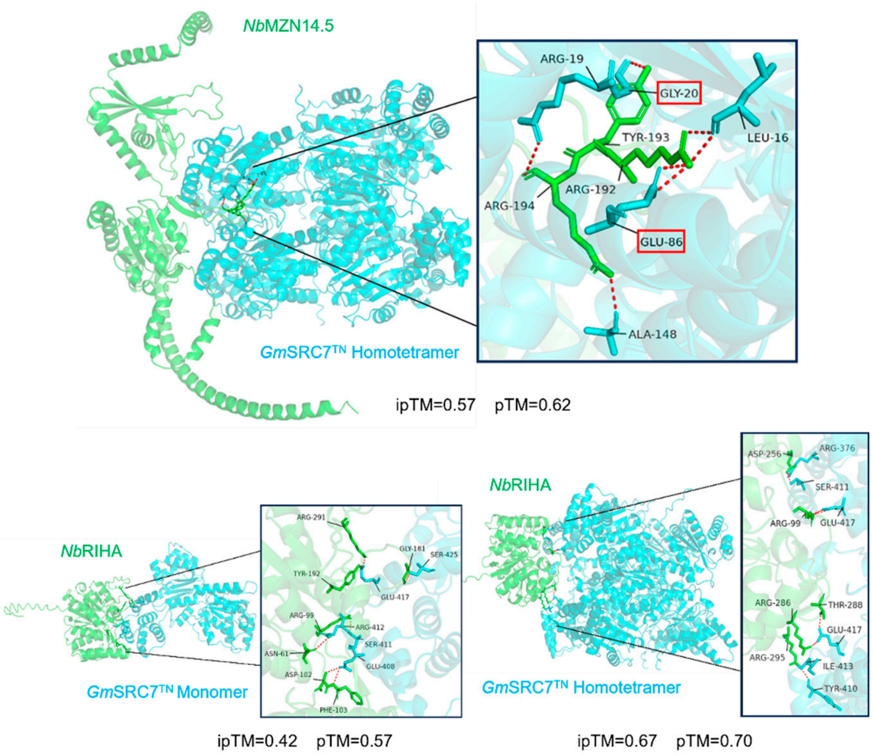

Figure S10 Structural modeling of interactions between GmSRC7^{TN} and its candidate interactors using AlphaFold3. Interaction models of GmSRC7^{TN} with candidate proteins (NbMZN14.5 and NbRIHA) are shown. Left panels display the overall interaction structures, and right panels highlight key interacting residues at the binding interface, including hydrogen bond interactions (red dashed lines). Predicted interaction confidence metrics (ipTM and pTM scores) are indicated below each model.
