# Supplementary material for: TurboID Screening and Functional Analysis of the Interactome of the Broad-Spectrum Antiviral Protein Soybean Resistance Cluster 7 (SRC7)
Source: Biology (Basel). 2026 Oct 29;15(21):1949. doi: 10.3390/biology15211949 (PMC13761510; doi:10.3390/biology15211949)
Supplement: Supplementary file 1 [file biology-15-01949-s001.zip › Table S1.pdf]

Table S1 The PCR primer sequences used in this study

Primers	Sequence (5′→3′)	Description
TN-F	gagaacacgggggacgagctcATGGCTGCAACAACACGTTCC	*SRC7^{TN}*
TN-R	agaggcacagtattgtctttTGTTTCCTGCTTTGTAGCATCAAAG	
NGFP-F	ccaaaaaagaagagaaaggtagaagaccccGTAGATCTGACTAGTAAAG	NLS-GFP
NLS-F	gagaacacgggggacgagctcATGCCAAAAAAGAAGAGAAAGGTAGAAG	
GFP-R	agaggcacagtattgtctttGCTAGCTTTGTATAGTTCATC	
TBo-F	AAAGACAATACTGTGCCTCTGAAGC	TurboID
TBo-R	CTTTTCGGCAGACCGCAGAC	
pQBF1	aaagcaggctcaggggatatcATGGTTACCCTCAAGAATCTCTTGC	*NbDOX1*
pQBR1	agctgggtgcagggcgatatcATGTTGAGGAATACGAAAGTAGAGTGG	
pQBF2	aaagcaggctcaggggatatcATGCAAAATTCTGAAAATCATCATCC	*NbRBOHC*
pQBR2	agctgggtgcagggcgatatcGTAAATTACCATAATGTATCCGAGGTTG	
pQBF3	aaagcaggctcaggggatatcATGGCTTTTCGTTTGAGTCATTT	*NbPER52*
pQBR3	agctgggtgcagggcgatatcCATAGAAGCCACAGAGCTGGGA	
pQBF4	aaagcaggctcaggggatatcATGGCTACTGACCACTCTGAAGATT	*NbPEX19*
pQBR4	agctgggtgcagggcgatatcCATAACACAGCAGTTCTGTTGGC	
pQBF5	aaagcaggctcaggggatatcATGGGGCAATCTTCATCTGCG	*NbMIJ24.8*
pQBR5	agctgggtgcagggcgatatcTCGATCTTCGCGACGAACTC	
pQBF6	aaagcaggctcaggggatatcATGATGATGATGCAAGATGTTTGG	*NbMZN14.5*
pQBR6	agctgggtgcagggcgatatcTTTGGCATTACCATTTTCCTTAGTA	
pQBF7	aaagcaggctcaggggatatcATGAAGAAGTCGAATTTTTTAAGAAGTT	*NbF6F22.26*
pQBR7	agctgggtgcagggcgatatcCTTGGGATTCATTCGAAGCG	
pQBF8	aaagcaggctcaggggatatcATGGATTCTTTCTTCAGCAAAGGT	*NbF23M19.10*
pQBR8	agctgggtgcagggcgatatcCTTATCGTGGGTGGACTTGATATTAT	
pQBF9	aaagcaggctcaggggatatcATGCTTGTTAGGATCAACACACTCC	*NbPALY*
pQBR9	agctgggtgcagggcgatatcACAGATAGGTAGAGGAGCACCATTC	
pQBF10	aaagcaggctcaggggatatcATGGCTGGTGTTGCACAAAAT	*NbPAL2/4*
pQBR10	agctgggtgcagggcgatatcACAGATTGGAAGAGGTGCACCA	
pQBF11	aaagcaggctcaggggatatcATGGCTACAATTGCAATTGAGTCA	*NbTBC5A*
pQBR11	agctgggtgcagggcgatatcCATCTCCGAGAGGAGGTTGCT	
pQBF12	aaagcaggctcaggggatatcATGCATGAAATTGACACACACGA	*NbF6N7.6*
pQBR12	agctgggtgcagggcgatatcTAGTACATCATCATCGTCCTGTTGATA	
pQBF13	aaagcaggctcaggggatatcATGGCACGTCCAAATGTGCC	*NbRIN4*
pQBR13	agctgggtgcagggcgatatcCCACAAAGGAAAGCAGCAGC	
pQBF14	aaagcaggctcaggggatatcATGGCAAGTTTAGTAGGAGGGGG	*NbPRPX1*
pQBR14	agctgggtgcagggcgatatcTTTCATGAAAGGACATTTTGAAGC	
pQBF15	aaagcaggctcaggggatatcATGGAAAGTATCGTAGGAGGAGGA	*NbPRPX2*
pQBR15	agctgggtgcagggcgatatcAATTGTCAAATTTTGGATAATTTTAAACG	
pQBF16	aaagcaggctcaggggatatcATGGATATTTTTCAGCAAAGACAGAA	*NbDRB4*
pQBR16	agctgggtgcagggcgatatcGCGATTTCGATTCAATTCTAAGC	
pQBF17	aaagcaggctcaggggatatcATGTCGAAATGTGATGGTGATTTG	*NbRIHA*
pQBR17	agctgggtgcagggcgatatcCATCAACTGTCCATGCAACTGAA	

pQBF18	aaagcaggctcaggggatatcATGTTTCAGGGTTTTTGGGATG	*NbVAD1*
pQBR18	agctgggtgcagggcgatatcTCCTTTCTGGTGCTTCCGTAGG	
pQBF19	aaagcaggctcaggggatatcATGGAGAACTTCCCAATTATCAACTT	*NbACO*
pQBF19	agctgggtgcagggcgatatcAGCAGTTGCAATAGGATCACATTT	
pQBF20	aaagcaggctcaggggatatcATGTTTGGGAGAGGACCAAAGA	*NbDNAJ*
pQBR20	agctgggtgcagggcgatatcACCTGTTCTGTATTCACATCATGTAATG	
qL1	GCAAGGCTAGTCACTTCTGC	*NbDOX1*
qR1	CCTCCAACATGCCCAAATGT	-qPCR
qL2	AACGCCCAATTCCTACTGGA	*NbRBOHC*
qR2	GCGGTAATCTCATCGAACCG	-qPCR
qL3	TGCTTTGTTGATGGTTGCGA	*NbPER52*
qR3	CAAGATACAGGCGTGTTGGG	-qPCR
qL4	TGGTTGGAGGACAACAAAGC	*NbPEX19*
qR4	TGGAGCAAGCTCATGAACAA	-qPCR
qL5	AAATGTTCTGGACGGGCAGT	*NbMIJ24.8*
qR5	CATCTGCACATCAACAGGCG	-qPCR
qL6	ACTTCGTGGCCATATTCGGA	*NbMZN14.5*
qR6	CGTTTAGCTTGTGTGCAGGA	-qPCR
qL7	AGTACCGCAGAGACTGACCA	*NbF6FF22.26*
qR7	GAAACTGAGGGCCTAACGCT	-qPCR
qL8	CAGCAGCTAACCTTGCTCAG	*NbF23M19.10*
qR8	GATGCGAGATTCCTGTTCCG	-qPCR
qL9	ACAATGGGTGCTAACGGTGA	*NbPALY*
qR9	GGATCATCAGCGTAGGCGAA	-qPCR
qL10	TCACCCCGGACAAATTGAGG	*NbPAL2*
qR10	CAATTTGAGGGCCAAGCCAC	-qPCR
qL11	ACGTTCTCGCGGTCTTTTCT	*NbPAL4*
qR11	CCTCAATTTGTCCGGGGTGA	-qPCR
qL12	CACAAGGAAGAAGAGCGCAA	*NbTBC5A*
qR12	TGGAGTTGGAGCAGACTCAG	-qPCR
qL13	GTAATGGAGCTGGTCGTGGA	*NbF6N7.6*
qR13	GAAGCAGCTACTCGTCCTGA	-qPCR
qL14	GGAGCTGGTCGTGGATCAAA	*NbRIN4*
qR14	TCGCCTGCTGAGAATTTGGA	-qPCR
qL15	TGCAGAGGCTGTTGAAGACT	*NbPRPX1*
qR15	TTTGCAGATTCTACCGGCGT	-qPCR
qL16	GGCCCGAAGGATCAGTTGAA	*NbPRPX2*
qR16	GCCACCACCAAGTTTTGCAT	-qPCR
qL17	CTGATGGGCAGTCTTCTGCT	*NbDRB4*
qR17	TCATCGCTGCAAGGCAAAAC	-qPCR
qL18	GAACCACGTGTTGCAGACTT	*NbRIHA*
qR18	CCGAGTACGACCACTCTCTT	-qPCR
qL19	GGATTGCCCTTGCACATGAA	*NbVAD1*
qR19	CCGTAGGGAATCAGCAGCTA	-qPCR

qL20	CCTCCTATGCGCCACTCTAT	*NbACO*
qR20	CCAAAGCTGGTGCTGGAAAT	-qPCR
qL21	AACTGTCCCTTTCCCGCAAT	*NbDNAJ*
qR21	TCATTGACGGGCCAAGTTGT	-qPCR
