# Supplementary material for: TurboID Screening and Functional Analysis of the Interactome of the Broad-Spectrum Antiviral Protein Soybean Resistance Cluster 7 (SRC7)
Source: Biology (Basel). 2026 Oct 29;15(21):1949. doi: 10.3390/biology15211949 (PMC13761510; doi:10.3390/biology15211949)
Supplement: Supplementary file 1 [file biology-15-01949-s001.zip › Table S2.pdf]

**Table S2** VIGS silencing fragment sequences used in this study

| **Name** | **Silencing fragment sequence (5′→3′, 300 bp)** |
|---|---|
| VIGS: *NbDOX1* | AAAACTGCTAGCGAGGAGAAATTTCGTGGACACAGGGAAACAATTTAACA<br>TGATCGCTGCTTCTTGGATACAGTTTATGGTTCATGATTGGATCGATCACTT<br>GGAAGATACTCAACAGATTGAGCTTCATGCACCTAAAGAAGTTGCAAGTC<br>AGTGTCCTCTCAAGTCCTTTAAGTTTAACAAAACCAAGGAAACTCCAACA<br>AACTTTTATGAAATCAAAACTGGTCACTTGAACAGGCGTACCCCATGGTGG<br>GATGGAAGCGTTATATATGGGAGCAATGCAGAGGTTTTGAAGAAAGT |
| VIGS: *NbRBOHC* | ATAGGTATGAATAAGGAGTCTAAGGAATTTGCTGTAGAGCTATATGATGCGC<br>TAGCTCGGAGGAGAAACATTACAACTGATTCCATTAACAAAGCACAGCTC<br>AAAGAGTTCTGGGATCAAGTGGCTGACCAAAGTTTTGATTCTCGCCTTCA<br>AACTTTCTTTGACATGGTTGATAAAGATGCTGATGGTAGAATCACAGAAGA<br>AGAAGTCAGAGAGATTATAGGCCTTAGCGCGTCTGCCAACAGGCTGTCAA<br>CAATCCAGAAACAAGCTGATGAATACGCAGCA ATGATCATGGAAGAG |
| VIGS: *NbPER52* | ATGGCTTTTCGTTTGAGTCATTTGAGCCTTGCCCTGAGCCTTTTGGCTCTTG<br>CACTTGCAGGTGTTGCCATATATAGGAACACTTATGAAGCCATGAGTAAGG<br>GTTTCCAAACACTTTCTCCAGAGTTGGATCTGCTGGAGTCAGCGGCCAGC<br>ATTTTAACCCTAAATAATAATAATGCTGAGAAAAATTCAGAGAGCAAGTTA<br>ACCCAACCATTATCTCCAACGGCATGCATCTTCTCCGCTGTTCAGGGAGTT<br>GTGAACAGTGCAATTGATAGAGAAAGGCGCATGGGAGCTTCTCTC |
| VIGS: *NbPEX19* | CTTAGGGATGATGTTGCCCGATAGGAATGCTAAGAAAAAGGGGAAGCAAA<br>CGGCTTCCGGTGAGTCAAAAGACTCGCACGTAGCTGAAGCTCTTGATAGG<br>CTTAGGGAGCAGACTAGAGAGGCTGTTAAAGGATTGGAATCAGTGGCCGG<br>GCCGAGACCTGGCTTAGATAACTTGGGGAATGATGCAATGATGGAGGAGT<br>GGGTTAAGCAGTTTGAGGAGCTTTCTGGATCTCAGGACATGGAGTCGATA<br>GTAGAGACCATGATGCAACAGCTTTTGTCAAAGGAAATCCTTCATGAACC |
| VIGS: *NbMIJ24.8* | ATGGGGCAATCTTCATCTGCGGAGCAAGTGTCGCCGGAGCAAAGAGAGGC<br>AGAATCTTTGGCAGCTTCAACTGGAGCTCTTCCTTCTCTACAAAAAGCTTT<br>CTCTCTCCTTTCTGATCCTCAAACTCACTCCATTCCCCTCCCTTCTCTTCAG<br>AAATGTTTTGGGTTCTCCTTTGAGGGATCAACAAGTGAAGAATCGACAGT<br>GCCGAAGGAAATTCCAGTTTTGTTGAGTCATTTGAGTTCTTCAATAGTTGA<br>CTTGTTTTTCTTGTCTGAAAAAGGAGGTGTTAGTTGGGTTGAGTTC |
| VIGS: *NbMZN14.5* | CCAAAAATTTAAAAATTCAAAAGATTATTATGCCAAGATTGGTAAGGCGTG<br>GAAGCGTGGTTATCTTCTTTATGGTCCTCCCGGGACAGCGGTTAAGGACAA<br>TACCGAGCTAAGAAAGTTATTGATAGACACTACAAGTAAATCTATAATTGTA |

| | |
|---|---|
| | ATAGAAGACATCGATTGTTCCCTTGACCTTACCGGTCAAAGAGAGGAGAA<br>GAAGAAAAAAGACGAGGAAGATAAAGAAAAAGATGAGAAAGATGCCATT<br>ATGAAAAAGATGAAAAAAGGAGAGGTGAAAGAGAAAAGTGAAGTAAC |
| VIGS:<br>*NbF6F22.26* | GTGCCTCCAAATGACTTGCTGAATGGATTCGGTAGTTTACAGAAAGCCGGT<br>AGCATCCATGAAGACCCTCCTCATTCTGAAGCTGCAGATGTGAGAACTCCA<br>CTTGATCACGGTAATAGGCCTAATGCATCTCCAAATATCCTAGAGCAGAATG<br>CGAGATCCTCACTGAGGACCCAAAACTTTAACTCTGCACATGGTGGTGGC<br>AGAGGCATAACACCATCTTCTAATTATCATCATGGAGTTGGTCCTTCAGGGT<br>TTAGAGATGAAGGGTTGGAAGGAGAACAA TCATTTCCTGCAGAT |
| VIGS:<br>*NbF23M19.10* | ATGGATTCTTTCTTCAGCAAAGGTTTCAAAGCTGCTAAATGTAAAACCCTA<br>TTGAAATTGACGATTCCTCGTATAAAACTACTGAGGAATCGTAGGGAGATG<br>CAGCTGAAGCAGATGAGAAAAGAAATTGCTAAGCTACTAGAGACTGGTCA<br>GGAAGCTACTGCTCGGATTCGGGTAGAGCATATAATAAGAGAAGAGAAAA<br>TGATGGCAGCACAGGAGATAGTCGAGCTATTCTGCGAGCTTATATCTGTTC<br>GTCTTCCAATTATTGAAGCACAAAGGGAATGTCCTCTAGATCTGAAA |
| VIGS:<br>*NbPALY* | AGAGAATACTTGTTCGCCTACGCTGATGATCCTTGCAGTTGCAACTACCCT<br>TTAATGCAGAAACTGAGACAAGTACTTGTTGATCATGCAATGAAGAATGGT<br>GAAAGTGAGAAGAATGTGAACAGCTCAATCTTCCAAAAGATTGGAGCTTT<br>CGAAGACGAATTGAAGGCTGTTTTACCTAAAGAAGTTGAGAGTGCAAGAG<br>CTGCATTGGAAAGTGGCAACCCTGCTATTCCTAACAGGATTACAGAATGCA<br>GATCGTATCCATTGTACAGGTTTGTGAGAAAGGAGCTTGGAACAGAA |
| VIGS:<br>*NbPAL2* | AACTCCTTGGGCTTAATCTCAGCTAGAAAAACAGCTGAAGCCGTGGACAT<br>CTTAAAGCTAATGTCATCCACATATCTAGTCGCACTTTGCCAAGCGATAGAC<br>TTGAGGCATTTGGAAGAAAATCTGAGGAATGCAGTCAAGAACACGGTGAG<br>CCAAGTCGCTAAGAGAACCTTGACGATGGGTGCCAATGGAGAACTTCATC<br>CATCAAGATTCTGCGAAAAGGACTTGCTTCGAGTCGTGGACAGGGAATAC<br>GTCTTTGCATACGCTGATGACGCCTGCAGCGCTAACTACCCACTGATG |
| VIGS:<br>*NbPAL4* | GTCATCCACATATCTAGTTGCACTCTGCCAAGCGATAGACTTGAGGCATTT<br>GGAAGAAAATCTGAGGAATGCAGTCAAGAACACGGTGAGCCAAGTCGCT<br>AAGAGAACCTTGACAATGGGTGCCAATGGAGAACTTCATCCATCAAGATT<br>CTGCGAAAAGGACTTGCTTCGAGTCGTGGACAGGGAATATGTCTTTGCATA<br>CGCTGATGACGCCTGCAGCGCCAACTACCCACTGATGCAGAAACTGAGGC<br>AAGTACTCGTCGACCACGCCTTACAAAATGGCGAAAATGAGAAGAACAC |
| VIGS:<br>*NbTBC5A* | ATCCTGAGCATGGTAGCTATTTCCAGACTCCTGGATGCCAAGCCATGTTGA<br>GGAGAATTCTACTACTTTGGTGCCTTAGACATCCAGAGTATGGTTACAGAC |

| | |
|---|---|
| | AAGGGATGCATGAACTGTTGGCCCCACTTCTCTATGTCCTTCAAGCTGATA CGGAGCAGCTTTCTGAAGTTAGGAATCTTTACGAGGATCATTTTGCTGACA AATTTGATGGGTTCTCATTTCATGAAAATGACTTGACATATAAATTTGACTT TAAAAAATTCTCGGAATCTGTGGAAGATGATAA TGGATCTCAGA |
| VIGS: *NbF6N7.6* | CATCTCAACTTTTTCGTGAAGCTCAGGGAGATGGTTTCGTTCCTAATGACA GGAGGAAGCAAGACCAAAAAACAAATGATTATCCTCGCATTGCTGAATCT AATGATGTTCAGAAGTTGCCTGAAGATGTTCAAGTGCCTGCAGATCCCTTG GAGTTTGACAAATTGTCACTAGTATGGGGAAAACCAAGGCAGCCTCCACT GGGAACAGAAGAGTGGTCCACTTTCCTGGACTGTGAAGGTAGAGTCACA GACTCGGAAGCAGTAAGAAAGAGGATATTTTATGGAGGAGTCGAACAAG |
| VIGS: *NbRIN4* | CACAACATTGCTAAATCGCCAATTCATCCAAATTCTCAGCAGGCGAAGATA TCAGGACGAGTAGCTGCTTCGCCTCAGCCTGATAGAGGAGCTGTAGTTCC AAAATTTGGCGAATGGGATGATAACGATCCTCAATCTGCAGAAAACTATTC TGAGGTTTTTAACAGAGTGCGAAATGAAAGGAATGAAAGGAACAGAGGC CCTGGAAATGTGCTAGGCACACCTGGTAGAACATCTTACAGTACCGAAAG GCATCAACGAAACGAAAAGCAAAAGAGCTGC TGCTTTCCTTTGTGGTGA |
| VIGS: *NbPRPX1* | AGATCTATATTTTTACGAGGATTCGCGATTGAAATCCTACATGTTTATTCAGG ACCACCAGAAATAGTGTACAAGTTTAGGCATTGGGGTTACATGGAAGGTCC ATTTAAAGGACATGCTGCTACTGGTGGACTTGTAGAGCTCTTTGGGATTGG CATTTTTGAATTGGACAAAGAGAGTAACAAGATCGTTAAGGCAGAGATGT TCTTTGATAGAGGAGAATTGCTTGGAGGCCTTGTGAAAGGAGAAAGCAAT GATGAATTGGAATTGGCTGCTTCAAAATGTCCT TTCATGAAATAA |
| VIGS: *NbPRPX2* | ATGGTCCACAAGACAGATCCTAATGACTTCAAAACTGTTGATCCAAAAATA TTCACAAAGAGTGTCAATGGCAGGAAAGGGTTGACACCAGAAGAAAATG CAAAACTTGGTGGTGGCTACAATACATTCCTTCAAACGTCATTGCCAGAGA GTGTTCGAGTCTACAACCCTGATGAGGAGACATTTGAATCATCACAGAAA GTTTTCAGATCCATATTTTTACGAGGATTCGCCATTGAAATCCTTCATGTTTA TTCAGGACCACCAGAAATTGTGTACAAATTTAGGCATTGGGGTCAC |
| VIGS: *NbDRB4* | ATGGATATTTTTCAGCAAAGACAGAATAACGTTGGACAACCTTCTCGTGTC AATGGAACTCCATTGGCTGGTTGGGAAGCCAATACCTCGAAAGGGATGCT TCATCTATACAAGAATCGACTACAACAGTATGCCCAAAAGCAAAATCTCAC TTTACCCGTGTATTCTTCTGAACTTGATGGCCCTCCGCACGCCCATCAGTTT AGGTCAAAAGTGACTATTGATGGAAAAACTTATGAAACTCAGGAGTTCTT CTCTACATTGAAGGAAGCTGAACATGCAGCTGCAAAAGTTGCTTTT |
| VIGS: | ATGTCGAAATGTGATGGTGATTTGGTTGATGGATTGGTGAATCCTAACAATA |

| | |
|---|---|
| *NbRIHA* | ACTCTTTTGCTATGCAACGTGAAAAGATCATCATTGATACTGATCCTGGCAT<br>TGATGACAGTATGGCAATACTAATGGCATTTCAGACACCGGGAGTTGAGAT<br>CGTAGGCTTGACTACGATCTTTGGCAATGTTACTACAAAAGACGCTACACG<br>TAATGCATTACTTTTGTGTGAAGCTGCAGGATATCCGGATGTTCCAGTCGCC<br>GAGGGCAGCCCTGAACCTCTGAAGGGAGGGGAGCCACGTGTT |
| VIGS:<br>*NbVAD1* | TTTCCCATTCTTATAGTGATCACCATTGCTGTGATCCTGCTCTTAATGCAGAT<br>GAGCATACTTGTGTTATTAGGTCGACCTCAGCACGTCCACGTGATACCTCA<br>AGGGGACTGCACAGGTAGCATGTATCGACTGGGAGATAGGGGTATAGACA<br>CACTAGCTTTTCTGGATAAACAGATTAGTCATCTCAAAGAGGAGATGGTTA<br>TGGTCGAAACACTGCTCGGCAAAATGCAGCAGGAGCATACACTTCTAAAC<br>ACGAAATTGAAAGAGTTTGAGCGCCTACGGA AGCACCAGAAAGGA |
| VIGS:<br>*NbACO* | GAGGTGATCACCAATGGGAAATACAAGAGTGTAATGCACAGAGTGATTAC<br>ACAAACAGACGGGACTCGGATGTCATTAGCTTCGTTTTATAATCCAGGAGG<br>TGATGCAGTAATATTTCCAGCACCAGCTTTGGTTGAGAAAGACGCAGAGG<br>AAAGCAAAGCAGTTTATCCAAAGTTTGTGTTTGATGATTATATGAAGTTATA<br>TGCTGGACTCAAGTTTCAAGCCAAAGAGCCAAGGTTTGAAGCCATGATCA<br>AGGCCATGGAAACTGGTGTGAAATGTGATCCTATTGCAACTGCTTAA |
| VIGS:<br>*NbDNAJ* | TCCCTTTCCCGCAATGTAATTTGCTCCAAGTGCAGTGGTAAAGGATCCAAG<br>TCTGGTGCTTCAATGAAGTGTTCTGGTTGTAAAGGTAGTGGTATGAAGGTT<br>TCAATTAGACAACTTGGCCCGTCAATGATCCAGCAAATGCAGCACCCTTGT<br>AATGAATGCAAGGGTACTGGAGAGACTATTGACGATAAGGATCGGTGCCC<br>TCAATGCAAAGGTGAAAAAGTGGTTCAGGAGAAGAAAGTCCTAGAAGTT<br>CATGTTGAAAAAGGCATGCAAAATGGACAGA AAATTACATTCCCCGGA |
| VIGS:<br>*GmSRC7* | ATGGCTGCAACAACACGTTCCCTTCCATTCATCTATGATGTGTTCCTCAGCT<br>TCAGAGGGGAAGACACACGCTATGGTTTCACTGGCAATCTATACAGGGCT<br>CTCTGTGAGAAGGGAATTCATACCTTCTTTGATGAAGAGAAGCTTCACGGT<br>GGAGATGAAATAACACCTGCACTTTCGAAGGCAATTCAAGAGTCCAGGAT<br>TGCTATTACTGTGCTTTCTCAAAACTATGCTTTTTCCTCATTTTGTTTAGATG<br>AACTTGTAACCATCCTTCACTGCAAGAGTGAAGGGCTGTTGGTT |
